# Effects of moderate doses of ionizing radiation on experimental abdominal aortic aneurysm

**DOI:** 10.1371/journal.pone.0308273

**Published:** 2024-08-01

**Authors:** Goran Riazi, Chloe Brizais, Imene Garali, Rida Al-rifai, Helene Quelquejay, Virginie Monceau, Guillaume Vares, Lea Ould-Boukhitine, Damien Aubeleau, Florian Gilain, Celine Gloaguen, Morgane Dos Santos, Hafid Ait-Oufella, Teni Ebrahimian

**Affiliations:** 1 Experimental Radiotoxicology and Radiobiology Laboratory (LRTOX), Institute for Radiobiological Protection and Nuclear Safety (IRSN), Fontenay-aux-Roses, France; 2 Université de Paris, Inserm U970, Paris-Cardiovascular Research Center, Paris, France; 3 Accidental Exposure Radiobiology Laboratory (LRACC), Institute for Radiobiological Protection and Nuclear Safety (IRSN), Fontenay-aux-Roses, France; 4 Medical Intensive Care Unit, Hôpital Saint-Antoine, AP-HP, Sorbonne Université, Paris, France; University of Missouri School of Medicine, UNITED STATES OF AMERICA

## Abstract

**Background:**

Exposure to ionizing radiation has been linked to cardiovascular diseases. However, the impact of moderate doses of radiation on abdominal aortic aneurysm (AAA) remains unknown.

**Methods:**

Angiotensin II-infused *Apoe*^*-/-*^ mice were irradiated (acute, 1 Gray) either 3 days before (Day-3) or 1 day after (Day+1) pomp implantation. Isolated primary aortic vascular smooth muscle cells (VSMCs) were irradiated (acute 1 Gray) for mechanistic studies and functional testing in vitro.

**Results:**

Day-3 and Day+1 irradiation resulted in a significant reduction in aorta dilation (Control: 1.39+/-0.12; Day-3: 1.12+/-0.11; Day+1: 1.15+/-0.08 mm, P<0.001) and AAA incidence (Control: 81.0%; Day-3: 33.3%, Day+1: 53.3%) compared to the non-irradiated group. Day-3 and Day+1 irradiation led to an increase in collagen content in the adventitia (Thickness control: 23.64+/-2.9; Day-3: 54.39+/-15.5; Day+1 37.55+/-10.8 mm, P = 0.006). However, the underlying protective mechanisms were different between Day-3 and Day+1 groups. Irradiation before Angiotensin II (AngII) infusion mainly modulated vascular smooth muscle cell (VSMC) phenotype with a decrease in contractile profile and enhanced proliferative and migratory activity. Irradiation after AngII infusion led to an increase in macrophage content with a local anti-inflammatory phenotype characterized by the upregulation of M2-like gene and IL-10 expression.

**Conclusion:**

Moderate doses of ionizing radiation mitigate AAA either through VSCM phenotype or inflammation modulation, depending on the time of irradiation.

## Introduction

The impact of ionizing radiation on human health represents a major concern for both populations and stakeholders in the nuclear field. While the dose-response relationship in high dose ranges follows a well-characterized linear model [1], the explanatory model for low to moderate doses of ionizing radiation still remains a matter of debate [2, 3]. Cardiovascular diseases (CVDs) remain the leading cause of death worldwide. According to the World Health Organization, 17.9 million people succumbed to CVDs, constituting 32% of all causes of death [4]. In the context of cardiovascular diseases, numerous studies have demonstrated that exposure to ionizing radiation aggravates these pathologies beyond 0.5 Gy [5–8]. Experimental studies conducted on rodents below this threshold yield more contrasting results. Mitchel et al. [9] showed that the effects of low and moderate doses of ionizing radiation were not dose-proportional in experimental atherosclerosis and slowed plaque progression. Ebrahimian *et al*. [10] and Le Gallic *et al*. [11] reported that low and moderate doses of chronic exposure reduced plaque size and enhanced atherosclerotic lesion stability in mice. Rey *et al*. [12] have shown that in *Apoe*^-/-^ mice, chronic low-dose and low-dose-rate irradiation induced a switch towards an anti-inflammatory and anti-oxidative phenotype. In contrast, Mancuso *et al*. [13] demonstrated that acute but not chronic exposure had pro-atherogenic effects with a 1.6-fold increase in murine plaque size in the abdominal aorta, associated with higher expression of MMP9, an enzyme involved in extracellular matrix (ECM) remodeling. All these studies yield diverse and sometimes contradictory results, emphasizing the need to contextualize findings regarding dose, dose rate, the nature of ionizing radiation, and the pathological context.

AAA is another common cardiovascular disease, essentially in the elderly population. AAA is defined by a focal and permanent dilation of the aortic wall, exceeding 50% of the diameter upstream and is associated with a risk of life-threatening dissection and/or rupture. The physiopathology of AAA is complex and still not fully understood. Several cellular and molecular actors are involved, leading to chronic inflammatory process and degradation of the medial layer of the vascular wall [14]. Three interdependent major processes occur during the initiation and development of AAA: inflammatory cell infiltration, degradation of the ECM, and loss of VSMCs.

To date, the impact of ionizing radiation on AAA development has never been studied. The aim of our study was to investigate the effects of low to moderate doses of ionizing radiation on AAA development and to identify the underlying cellular and molecular actors. Using an experimental mice model of AAA with AngII infusion in *Apoe*^*-/-*^ mice, we showed that a single dose of X-rays (1 Gy) before and after Ang II infusion limited AAA development through different mechanisms. Ionizing radiation impacted on VSMC, and immune cells and led to an increase in collagen content in the aortic wall.

## Materials & methods

### Animals

All experiments and procedures were conducted in accordance with the Guide for the Care and Use of Laboratory Animals, as published by the French regulations for animal experiments (Ministry of Agriculture Order No. B92-032-01,2006) following European Directives (86/609/CEE), and were approved by the local ethical committee of the Institute for Radiological Protection and Nuclear Safety (Permit Numbers: P19-21, P20-07, P21-09, P21-19). Male *Apoe*^*-/-*^ mice on C57Bl/6J background, aged between 6- and 8-weeks, were obtained from the Charles River Laboratory.

#### AngII-induced AAA

AngII (Sigma-Aldrich) reconstituted in PBS at a concentration of 25 mg/mL was infused in 8- to 10- week-old mice via subcutaneous osmotic pumps (Alzet model 2004, DURECT Corp, Cupertino, CA 95014) releasing a constant concentration of 1,000 ng.kg^-1^.min^-1^ for either 7 or 28 days. The pumps were set up under aseptic conditions after isoflurane anesthesia (3% for induction and 1.5% for maintenance). A solution of 100 μL of Buprenorphine diluted in NaCl 0.9% (1 mg.kg-1) was injected subcutaneously 15 minutes before surgery. For model validation, 6 mice received only PBS infusion. The mice in each group were divided into two subgroups: one subgroup was euthanized on day 7 (D7), and the second subgroup was euthanized on day 28 (D28). Mice were killed by cervical dislocation under isoflurane anesthesia.

### VSMC culture

The cell culture involved primary VSMC obtained from the aortas of male C57BL/6J mice aged between 6- and 8-weeks (Charles River). The mice were anesthetized through intraperitoneal injection of a ketamine solution (Ketamine 500, Virbac) and xylazine (Rompun 2%, Bayer) in 0.9% NaCl. The aortas were subsequently removed and washed in culture medium. The culture medium consisted of DMEM F12 (D0697, Sigma-Aldrich) with 20% fetal bovine serum (FBS) (A5256701, Gibco), supports VSMC proliferation while not conductive to endothelial cell growth [15]. The aortas were then digested for 7 minutes and 30 seconds in a freshly prepared enzymatic solution at 37°C/5% CO_2_. The solution comprised 20 mL of Hanks’ Balanced Salt Solution (HBSS), 20 mg of Collagenase II (17101015, Gibco), 200 μL of Penicillin/Streptomycin (15070063, Gibco), 100 μL of Fungizone (15290018, Gibco), and elastase (E125, Sigma-Aldrich). The *adventitia* was delicately dissected under an anatomical microscope to retain only the *intima* and *media*. The *intima* and *media* were then cut into pieces and subjected to a second digestion for 1 hour with gentle agitation at 37°C/5% CO_2_. The suspended cells were centrifuged at 1500 rpm for 5 minutes, after which the supernatant was then removed and replaced with fresh culture medium. The cells were transferred to a fibronectin coated T25 flask (diluted 1/10) and incubated at 37°C/5% CO_2_ for 7 days without disturbance. The culture medium was changed every 48 hours, and cell passage was performed when they reached 70% confluence.

### Irradiation platform

All irradiation was performed on the Small Animal Radiation Research Platform (SARRP) from XSTRAHL (XSTRAHL Ltd., Camberley, UK) at IRSN (Fontenay-aux-Roses, France). The SARRP is an image-guided micro irradiator. It consists of a Varian X-ray tube mounted on a gantry and is primarily designed for the irradiation of small animals and cells culture. For both *in vivo* and *in vitro* irradiation, the irradiation parameters were: a voltage of 220 kV, a current intensity of 5 mA, an inherent and additional filtration of 0.8 mm of Be and 0.15 mm of Cu respectively, and a half value layer of 0.667 mm of Cu. Reference dosimetry measurements were performed with the 31010 cylindrical ionization chamber with a cavity volume of 0.125 cm^3^ calibrated in dose to water (mouse irradiation) or air kerma (cell irradiation).

### Animal irradiation

The mice were anaesthetized with a combination of intraperitoneally injected 300μL of ketamine/xylazine before undergoing total-body irradiation. During irradiation, the mice were positioned in the open field of the radiation chamber at a 75.5 cm source-to-target distance. As the mice were irradiated in threes, dosimetry measurements were performed in the same conditions using three homemade mouse phantoms (water equivalent) and the ionization chamber was inserted into the central mouse phantom. The administered dose was 1 Gy in dose to water at a dose rate of 0.25 Gy.min^-1^ as a single dose, with a relative dose uncertainty of 5%. The mice were divided into three groups based on the irradiation procedure. The first group served as a non-irradiated control, the second group underwent irradiation 3 days before AngII supplementation (D-3 IR), and the third group underwent irradiation 1 day after AngII supplementation (D+1 IR). The mice in the control group received the same dose of ketamine/xylazine on the days of irradiation.

### Cell irradiation

The cells were irradiated in their culture medium using the SARRP 48 hours after being FBS-starved (FBS 0.5%). Irradiation was performed with the cells positioned in the openfiled of the radiation chamber (at a 75.5 cm source-to-target distance) following the protocol described in Dos Santos *et al* [16]. The administered dose was 1 Gy in water kerma at a dose rate of 0.25 Gy.min^-1^ as a single dose. The relative dose uncertainty was 5%. The cells were irradiated in 96-well plates for cell migration and proliferation assays, as well as for Bioplex analysis. The wells were filled with 100 μL of cell culture medium. T25 flasks were used for gene expression studies. The T25s were filled with 5 mL of cell culture medium.

### Dilation measurement

Aortic dilation was assessed using a high-frequency ultrasound system, Vevo 3100 (VisualSonics, FUJIFILM) with a high-frequency transducer (MX550D: 25–55 MHz). Mice were shaved at the abdominal level and anaesthetized with isoflurane (1.5%) for image acquisition. Measurements were taken from cross-sectional images of the suprarenal abdominal aorta following the protocol described in Sawada *et al* [17]. Evaluations were conducted prior to AngII infusion as a control, on D7 and on D28. Data was analyzed with VevoLab 5.5.1 (VisualSonics, FUJIFILM).

### Blood pressure measurement

Systolic blood pressure was quantified using a Tail Cuff CODA^®^ Non-Invasive Blood Pressure System 4.1 (Kent Scientific Corporation, USA), a non-invasive method for measuring blood pressure at the tail of rodents. To enhance measurement reproducibility, all readings were taken in the morning at the same time after a 3-day acclimatization period. The systolic blood pressure of the animals was measured before irradiation and AngII supplementation as a control, and on D21. Each measurement session comprised 20 cycles of volume/pressure, with the initial 5 cycles disregarded for analysis.

### Blood, heart, and aorta sampling

The mice were terminally anaesthetized by intraperitoneal injection of ketamine/xylazine. The blood was collected by cardiac puncture with a heparinized syringe. It was centrifuged at 1000g for 10 minutes to recover the plasma which was stored at -80°C. The hearts were weighed using a precision balance immediately after being harvested. Abdominal aortas were collected. A portion of the abdominal aortas was rapidly frozen in liquid nitrogen and stored at -80°C, while another portion was promptly embedded in Tissue-Tek O.C.T. Compound (Sakura OCT 4583) for frozen sections and stored at -80°C.

### Histology and immunohistochemistry

The mice sacrificed on D7 had their abdominal aortas frozen in O.C.T. The aortas were transversely sectioned at a thickness of 7 μm using a cryostat and stored at -80°C for subsequent histological and immunohistological analyses.

The sections were stained with Orcein to visualize elastin layers. The average number of elastin layers was quantified by a researcher blinded to the experimental protocol, involving 4 measurements per section and 8–10 sections per mouse.

Immunohistochemistry for VSMC utilized an anti-Rabbit polyclonal *α-smooth muscle actin* (α-SMA) primary antibody (ab5694, Abcam) revealed with a Donkey anti-Rabbit secondary antibody (A32795, ThermoFisher). The percentage of cellular area positive for *α-SMA* staining in the *media* was quantified using Histolab software (Microvision).

Collagen I staining employed an anti-Rabbit *Col1a1* primary antibody (234167, Sigma-Aldrich) revealed with a Donkey anti-Rabbit secondary antibody (A32795, ThermoFisher). The total surface area positive for *Col1a1* (collagen type I fiber) in the entire aorta was quantified using Histolab software.

For the identification of macrophages and resident aortic macrophages, sections were stained with an anti-Rat *CD68* (ab53444, Abcam) combined with an anti-Rabbit *Lyve-1* primary antibody (ab33682, Abcam) revealed with Goat anti-Rat (A11007, ThermoFisher) and with Donkey anti-Rabbit secondary antibodies (A32795, ThermoFisher). The percentage of *Lyve-1* and *CD68*-positive cells in the entire aorta and the percentage of *CD68*-positive cells in the entire aorta for macrophages and resident macrophages respectively was quantified using QuPath-0.4.0.

### Quantitative real time-polymerase chain reaction

Total RNA from the aortas was extracted using the RNeasy kit (Qiagen). RNA quality (260/280 nm) was determined with a Nanodrop ND 1000 spectrophotometer. The reverse transcription was performed with the high-capacity cDNA Reverse Transcription Kit from Applied Biosystems (Life Technologies).

Quantitative PCR analysis was performed with a QuantStudio 12K Flex Real-Time PCR System (Life Technologie) using Taqman 6 carboxyfluorescein-labeled probes from ThermoFisher (Tgf-β R1, Mm00436964_m1; Prkg1, Mm01207548_m1; Fbn1, Mm00514908_m1; Lama5, Mm01222029_m1; Tpm2, Mm00437172_g1; Col1a1, Mm00801666_g1; Acta2; Mm00725412_s1) and Sybr green with a standard thermal cycler protocol (50°C for 2 minutes, 95°C for 15 minutes and 60°C for 1 minute repeated 45 times). All samples were run duplicates and normalized to Gapdh, Hprt, β-actin or Ywhaz. The following primer sets were used: Gapdh (Forward 5’- AGGTCGGTGTGAACGGATTTG -3’, Reverse 5’- TGTAGACCATGTAGTTGAGGTCA -3’); b-actine (Forward 5’- AGGAAGGAAGGCTGGAAGAG -3’, Reverse 5’- TCCCTGGAGAAGAGCTACGA -3’); Mmp-2 (Forward 5’- TTCAGGTAATAAGCACCCTTGAA -3’, Reverse 5’- TAACCTGGATGCCGTCGT -3’); Mmp-9 (Forward 5’- CGGCACGCCTTGGTGTAGCA -3’, Reverse 5’- AGGTGAGGGGGCGCCTGTAG -3); Mmp-12 (Forward 5’- TTTCTTCCATATGGCCAAGC -3’, Reverse 5’- GGTCAAAGACAGCTGCATCA -3’; Mmp-13 (Forward 5’- CCTTCTGGTCTTCTGGCACAC -3’, Reverse 5’- GGCTGGGTCACACTTCTCTG -3’); Timp1 (Forward 5’- GATATGCCCACAAGTCCCAGAACC -3’, Reverse 5’- GCACACCCCACAGCCAGCACTAT -3’); Timp2 (Forward 5’- CCCCTCCACCGTTCCTCTCTTTTC -3’, Reverse 5’- TCACCCAGCCAGCACCTCACC -3’); Timp3 (Forward 5’- CCTGGCTATCAGTCCAAAC -3’, Reverse 5’- GTTGCTGATGCTCTTGTCT -3’); Il-4 (Forward 5’- AACTGTGGGCTGAGCACAGACA -3’, Reverse 5’- GCGCATCGCCTTCTATCGCCTTC -3’); Il-6 (Forward 5’- CCTTCTTGGGACTGATGCTGGTG -3’, Reverse 5’- AGGTCTGTTGGGAGTGGTATCCTC -3’); Il-10 (Forward 5’- AAGGCAGTGGAGCAGGTGAA -3’, Reverse 5’- CCAGCAGACTCAATACACAC -3’); Tnf-a (Forward 5’- AGCCGATGGGTTGTACCTTG -3’, Reverse 5’- GTGGGTGAGGAGCACGTAGTC -3’); Tgf-b (Forward 5’- GGCTACTTTGCCAACTACTGC -3’, Reverse 5’- CTGCTCCACCTTGTGTTGC -3’); Ccl2 (Forward 5’- CCCCACTCACCTGCTGCTA -3’, Reverse 5’- TACGGGTCAACTTCACATTCAAA -3’); Ccr2 (Forward 5’- AAGAGGGCATTGGATTCACCACAT -3’, Reverse 5’- ATGCCGTGGATGAACTGAGGTA -3’); Lyve1 (Forward 5’- TGGTGTTACTCCTCGCCTCT -3’, Reverse 5’- TTCTGCGCTGACTCTACCTG -3’); Retnla (Forward 5’- GGAGCTGTCATTAGGGACATCA -3’, Reverse 5’- TCCCAAGATCCACAGGCAAA -3’); Chi3l3 (Forward 5’- TCTGGGTACAAGATCCCTGAA -3’, Reverse 5’- TTTCTCCAGTGTAGCCATCCTT -3’). Relative expression was calculated using the ΔΔCt method.

### Plasma cytokine expression (Bioplex)

The cytokine expression was measured with the Bio-Plex Pro Mouse Cytokine assay kit (#M60009RDPD, BIO-RAD), containing fluorescent microspheres conjugated with monoclonal antibodies specific to IL-1α, IL-1β, IL-2, IL-3, IL-4, IL-5, IL-6, IL-9, IL-10, IL-12 (p40), IL-12 (p70), IL-13, IL-17A, IFN-γ, and TNF-α (N = 9 per condition).

Twenty microliters of undiluted plasma were used per sample in duplicate. The results were read using a Bio-Plex 200 system (BIO-RAD). Data was analyzed with Bio-Plex Manager software.

### Cell proliferation

Cell proliferation was measured using the WST-1 kit (Abcam, Quick Cell Proliferation Assay, ab65475). Cells were cultured in 96-well plates at a concentration of 35,000 cells per well, with 100 μL of cell culture medium. Wells without cells were used as blanks. All measurements were performed in duplicate. Ten microliters of the WST-1 reagent were added to each well. The plates were then incubated for 2 hours at 37°C/5% CO2. The plates were shaken for 1 minute in an orbital shaker at an amplitude of 1 mm before measuring absorbance. Absorbance was measured with a spectrophotometric plate reader (Infinite 200 Pro, Tecan) at 440 nm.

### Cell migration

Cell migration was assessed using a scratch assay. Cells were cultured in 96-well plates at a concentration of 40,000 cells per well with 100μL of cell culture medium. A scratch was made at the center of each well using a woundmaker^®^. The wells were washed twice with culture medium. Cells were then incubated in an Incucyte^®^ Live Cell Analysis system at 37°C/5% CO2 for 96 hours. Photos of each well were taken every 2 hours to analyze the time taken by the cells to repopulate the scratch area. All measurements were performed in duplicate.

### RNA-sequencing

Total RNA was extracted from abdominal aortas using a RNAEasy Kit (Qiagen), then mRNA was selected by polyA selection. RNA-sequence libraries were prepared with the TruSeq Stranded mRNA Prep Kit (Illumina, San Diego, CA, USA). Sequencing was performed at Genewiz Azenta Life Sciences (Leipzig, Germany) on a NovaSeq 6000 machine (Illumina) to generate at least 20 million reads. After quality control using FastQC, the reads were mapped to the Ensembl GRCm38 reference mouse genome with the STAR aligner software (v2.7.9a) using the quantMode GeneCounts option to generate read counts. Analysis of differential gene expression was performed with the edgeR package (v3.36.0, using Bioconductor v1.30.18 on R v4.1.0), with TMM normalization [18] and GLM analysis (no intercept) with quasi-likelihood F-tests [19].

A major goal of RNA-seq analysis is to identify differentially expressed genes and to infer biological meaning for further studies. Unfortunately, in our case, the differential gene expression (DGE) analysis did not produce good results (S1 Fig), due to the small cohort of samples and the specificity of the LDR. For this reason, we applied Principal Component Analysis (PCA) and Partial Least-Squares Discriminant Analysis (PLS-DA) [20] to descriptive statistics analysis PLS-DA is a multivariate dimensionality-reduction analysis. It can be considered as a supervised version of PCA. In contrast with PCA, PLS-DA finds the projection best suited to distinguish between levels of the target variable. The results are shown in (S2 Fig), with between 5 to 7 mice used per group. PLS-DA outperformed PCA. This is due to the strong correlation between the RNA-seq features for class members (Control, D+1 IR, D-3 IR) that PLS-DA can detect. In our study, PLS-DA with feature selection process (sPLA-DA) was performed in accordance withmixOmics package instructions. We used a Pathway Enrichment Analysis (PEA) as Metascape [21], to validate and evaluate the biology of the features (genes) selected.

### Statistical analysis

Statistical analysis was performed for Pearson’s Chi-squared test and nonparametric Kruskal-Wallis one-way ANOVA, Wilcoxon rank-sum test, and Mann-Whitney test.

## Results

### Irradiation reduces AngII-induced aortic dilation and protects against AAA development

**Fig 1:**
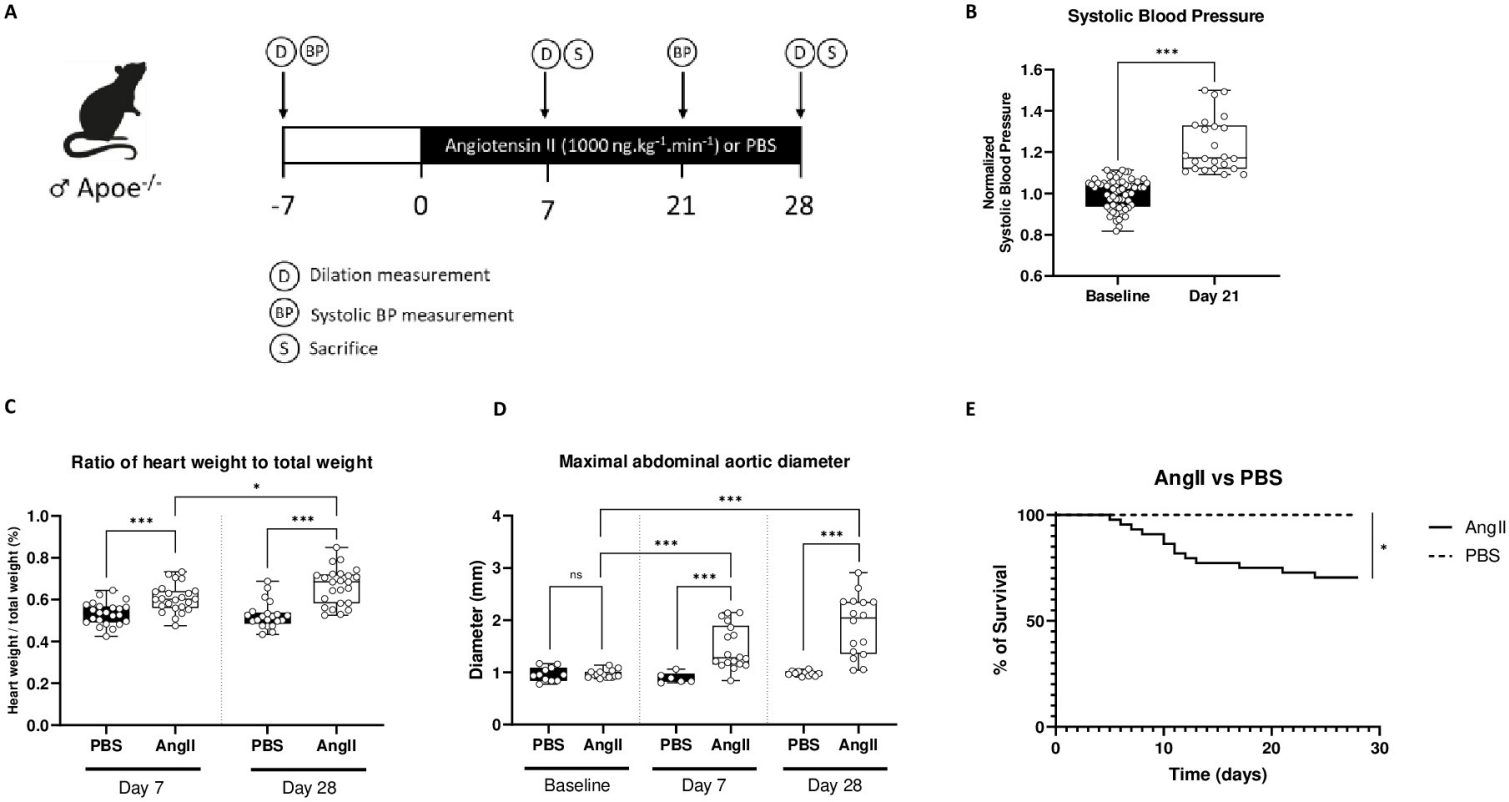
Validation of experimental model for inducing AAA by AngII. A, Schematic representation of the validation of the AAA induction model in Apoe-/- mice receiving AngII or PBS during 7 days or 28 days (in each group, N = 9). B, Systolic BP in mice receiving AngII, normalized to baseline data (N = 45 to 89 mice). C, Ratio of heart weight relative to the total weight at D7 and D28 in PBS-treated mice and AngII-treated mice. D, Maximal abdominal aortic diameter at baseline, D7 and D28 in PBS-treated mice and AngII-treated mice. E, representative images of a cross-sectionnal view PBS-treated mice aorta [1] and AngII-treated mice aorta [2] at D28. F, representative picture PBS-treated mice aorta [1] and AngII mice aorta [2] at D28. G, 28-day survival curve comparison between PBS-treated mice and AngII-treated mice. *P≤0.05, **P≤0.01 and ***P≤0.001.

First, we validated the experimental model of AAA induced by AngII infusion as described in the Materials & Methods section (Fig 1A). As expected, AngII infusion increased systolic blood pressure whereas PBS infusion did not (Fig 1B). AngII infusion also led to an increase in heart weight at both day 7 and day 28 (Fig 1C). Abdominal aortic diameter was similar at baseline between groups. Aorta diameter remained unchanged over time in mice receiving only PBS while AngII infusion induced a significant increase in abdominal aortic diameter at D7 and D28 (Fig 1D–1F). Finally, Day-28 mortality due to the dissecting aneurysm was 42% in the Angio II group. No death was observed during the follow-up in mice receiving PBS (Fig 1F).

**Fig 2:**
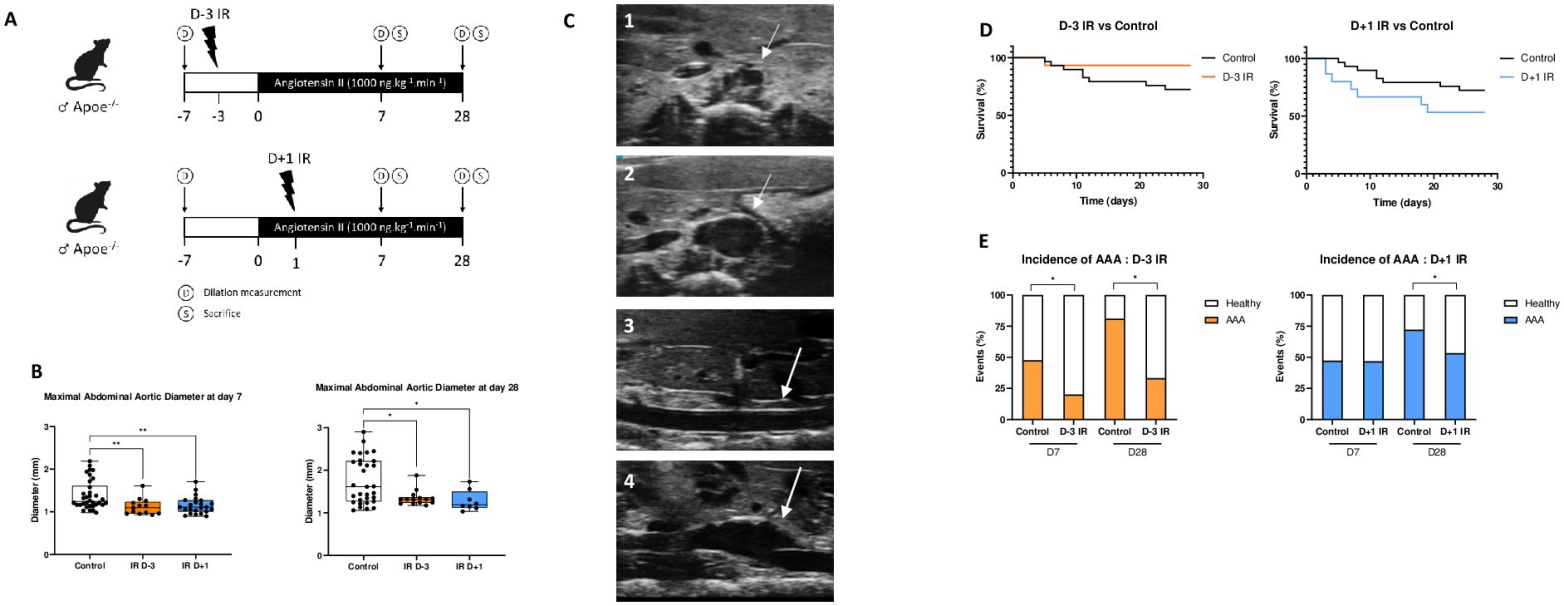
**A**, schematic representation of mice’s irradiation procedures, D-3 IR (top), where mice are irradiated 3 days before AngII supplementation begins, and D+1 IR (bottom), where mice are irradiated 1 day after the start of AngII supplementation (N = 9 to 14 mice per group). **B**, Maximal abdominal aortic diameter at D7 (**left**) and D28 (**right**) compared to the non-irradiated control. **C**, representative images of a cross-sectionnal view healthy abdominal aorta [1] and an aneurysmal abdominal aorta [2] and a longitudinal view of healthy abdominal aorta [3] and an aneurysmal abdominal aorta [4] using ultrasound. **D**, 28-day survival curve of the D-3 IR (**left**) and D+1 IR (**right**) procedures compared to the non-irradiated control (N = 9 to 14 mice per group). **E**, quantification of the incidence of AAA (diameter ≥ 50% of baseline diameter + death due to rupture) at D7 and D28 in mice in D-3 IR (left) and D+1 IR (right) procedures compared to the non-irradiated control (N = 9 to 14 mice per group). *P≤0.05 and **P≤0.*01*.

To investigate the effects of irradiation on the development of AAA, we tested two irradiation procedures. First, mice were irradiated at 1 Gy 3 days before AngII infusion (Day-3 IR) in a non-pathological context, and second, irradiation was done 1 day after AngII supplementation (Day+1 IR) in pathological condition. One group of mice was sacrificed at day 7 for tissue analyses, and another at day 28 for survival curve analysis and disease development Measurements of aorta diameter were performed at baseline before irradiation, at day 7, and at day 28 (Fig 2A). Regardless of the procedure, irradiated mice exhibited a reduction in aortic dilation at both day 7 (**left**) and day 28 (**right**) compared to non-irradiated control mice (Fig 2B and 2C). However, there was no difference in mortality between the irradiated and non-irradiated groups (Fig 2D). The global incidence of AAA differed with the time of irradiation. Only irradiated mice in the Day-3 group showed a decreased AAA incidence compared to the non-irradiated control group at day 7. At day 28, both Day-3 IR and Day+1 IR groups exhibited a significant reduction in AAA incidence compared to the non-irradiated control group (Fig 2E).

### Irradiation modulates AngII-induced vascular wall remodeling

**Fig 3:**
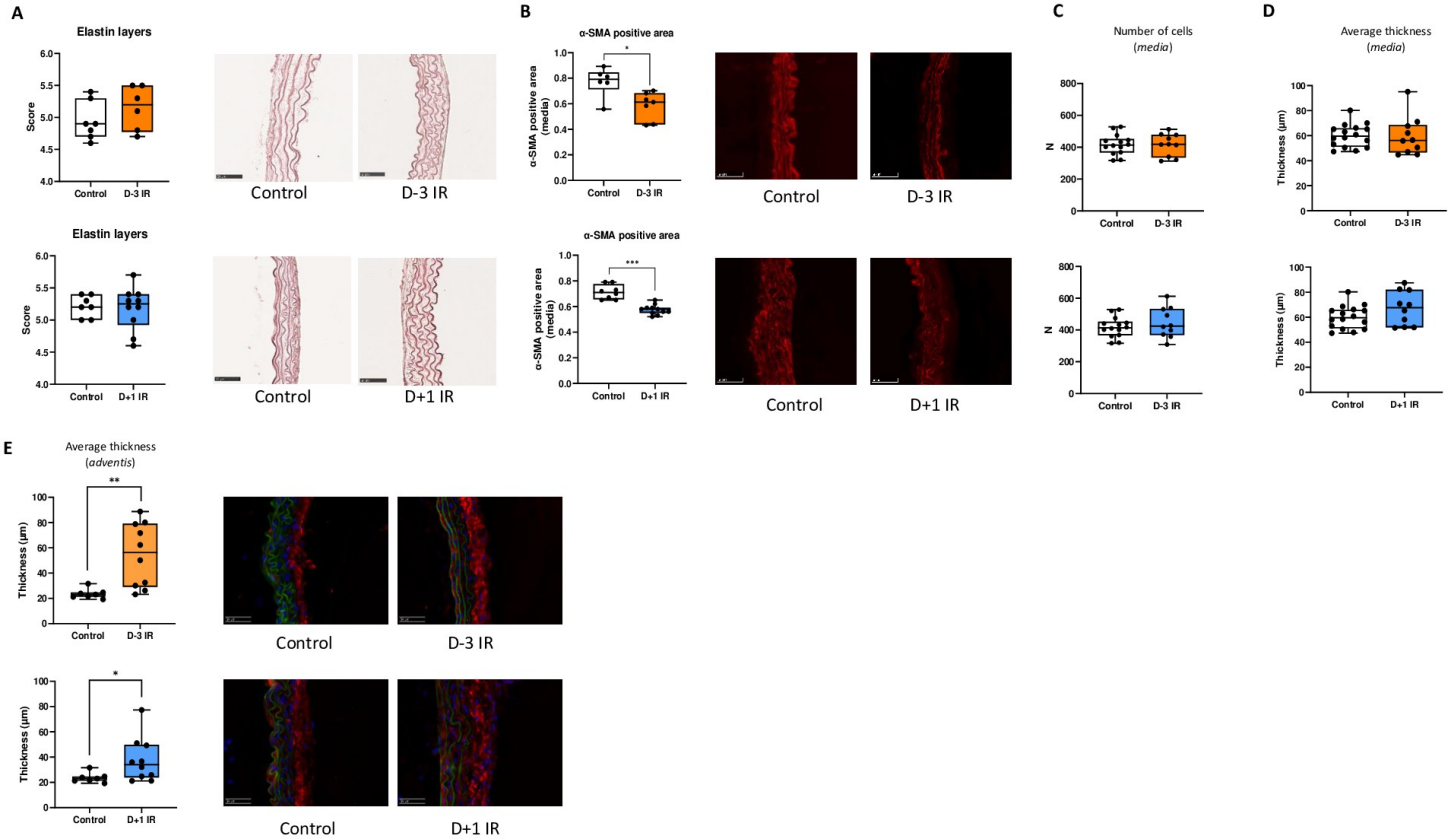
**A**, quantification of the average number of elastin layers in the media after Orcein staining at D7 in D-3 IR (**top**) and D+1 IR (**bottom**) procedures (N = 6 to 10 mice per group). **B**, quantification of α-SMA positive area in the media at D7 in D-3 IR (**top**) and D+1 IR (**bottom**) procedures (N = 6 to 11 mice per group). **C**, quantification of the number of cells in the media at D7 in D-3 IR (**top**) and D+1 IR (**bottom**) procedures (N = 10 to 14 mice per group). **D**, quantification of the average thickness in the media at D7 in D-3 IR (**top**) and D+1 IR (**bottom**) procedures (N = 10 to 16 mice per group). **E**, quantification of the average thickness in the adventitial tunic at D7 in D-3 IR (**top**) and D+1 IR (**bottom**) procedures (N = 10 to 16 mice per group). *P≤0.*05*, **P≤0.01 and ***P≤0.*001*.

Next, we investigated the impact of irradiation of vascular wall structure. After Orcein staining and morphometry analysis, we found that irradiation has no impact on the number of elastin layers at day 7 (Fig 3A). *α-SMA* immunostaining showed a significant decrease in actin content in the *media* of mice from both Day-3 and Day+1 irradiated groups (Fig 3B). Of note, the number of cells in the aortic wall was similar between irradiated and non-irradiated groups (Fig 3C) as well as the media thickness (Fig 3D). Finally, we quantified the collagen I content on the adventitia after immunostaining. As illustrated in Fig 3E, irradiation at Day-3 or Day+1 was associated with a significant increase in collagen content when compared to the non-irradiated control group.

### Irradiation modulates VSMC phenotype

**Fig 4:**
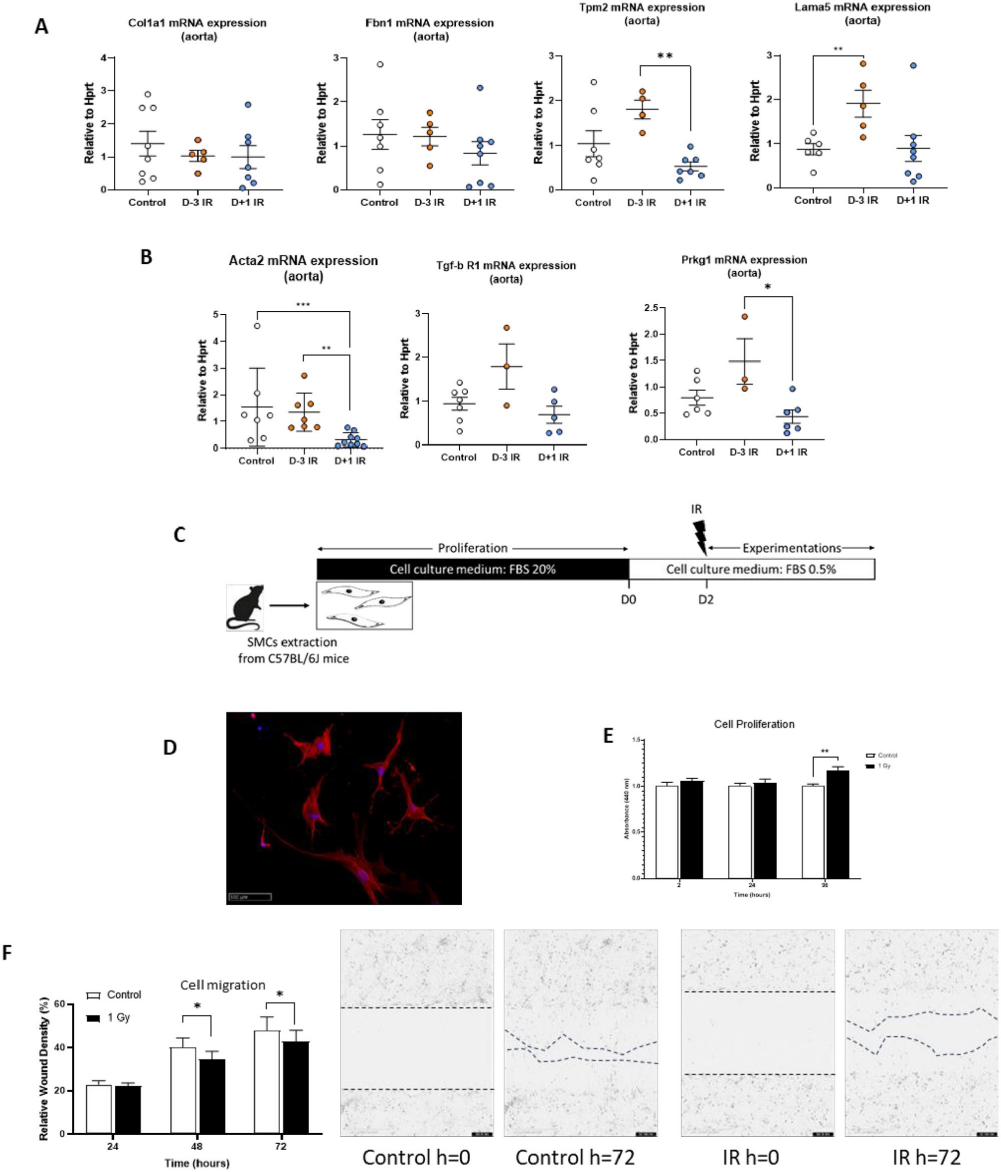
**A**, Quantification of *Col1a1*, and *Fbn1* mRNA expression in the aorta at day 7 by RT-qPCR (SYBR) (N = 4 to 8 mice per group). **B**, Quantification of *Acta2*, *Lama5*, *Tgf-β R1*, and *Prkg1* mRNA expression in the aorta at day 7 by RT-qPCR (SYBR) (N = 3 to 8 mice per group). **C**, Schematic representation of cell’s irradiation procedure. **D**, Immunostaining with α-SMA (red) and DAPI (blue) antibody of SMC derived from mouse aortas (magnification x20). **E**, Cell proliferation kinetics from 2 to 96 hours post-irradiation conducted performed with a WST-1 assay. **F**, Cell migration kinetics measured by relative wound density from 0 to 96 hours post-irradiation performed using a scratch test. *P≤0.*05*, **P≤0.01 and ***P≤0.*001*.

The decrease in alpha-actin content within the media after irradiation, despite a non-change in cell number in the media suggested a phenotypic switch of VSMC with loss of contractile profile. To evaluate our hypothesis, we analyzed gene expression in the abdominal aorta with qPCR. We found modifications of transcriptomic signature mainly in the Day-3 irradiated group. Indeed, we observed an increase in *Tpm2* and *Lama5 transcripts*, coding for tropomyosin and laminin respectively. *Tpm2* was elevated in comparison with the non-irradiated control group, and *Lama5* was elevated in comparison with the Day+1 irradiated group. Gene expression of *Col1a1* and *Fbn1*, coding for Collagen 1 and Fibronectin respectively, remained unchanged regardless of the irradiation group (Fig 4A). We also analyzed the gene expression related to contractile phenotype. We found a significant reduction in *Acta2* mRNA levels, which codes for actin, in Day+1 irradiated group compared to the two other groups. *Prkg1*, coding for a cGMP-dependent protein kinase involved in cellular contractility, was reduced in the Day-3 group compared to the Day+1 group. (Fig 4B). To further investigate the impact of irradiation on VSMC phenotype we performed additional *in vitro* experiments on primary VSMCs exposed to irradiation and Ang II (Fig 4C). *α-SMA* immunostaining confirmed the VSMC contractile phenotype at baseline (Fig 4D). Using WST-1 kit, we showed that irradiation significant increased proliferative capacity of VSMC when compared to non-irradiated cells (Fig 4E). In addition, VSMC migration following a scratch test was significantly reduced after irradiation (Fig 4F). Altogether, these results highly suggested that irradiation modulated VSMC phenotype with a switch toward a less contractile profile, essentially when irradiation was done before AngII infusion.

### Irradiation modulates inflammatory responses within the vascular wall

**Fig 5:**
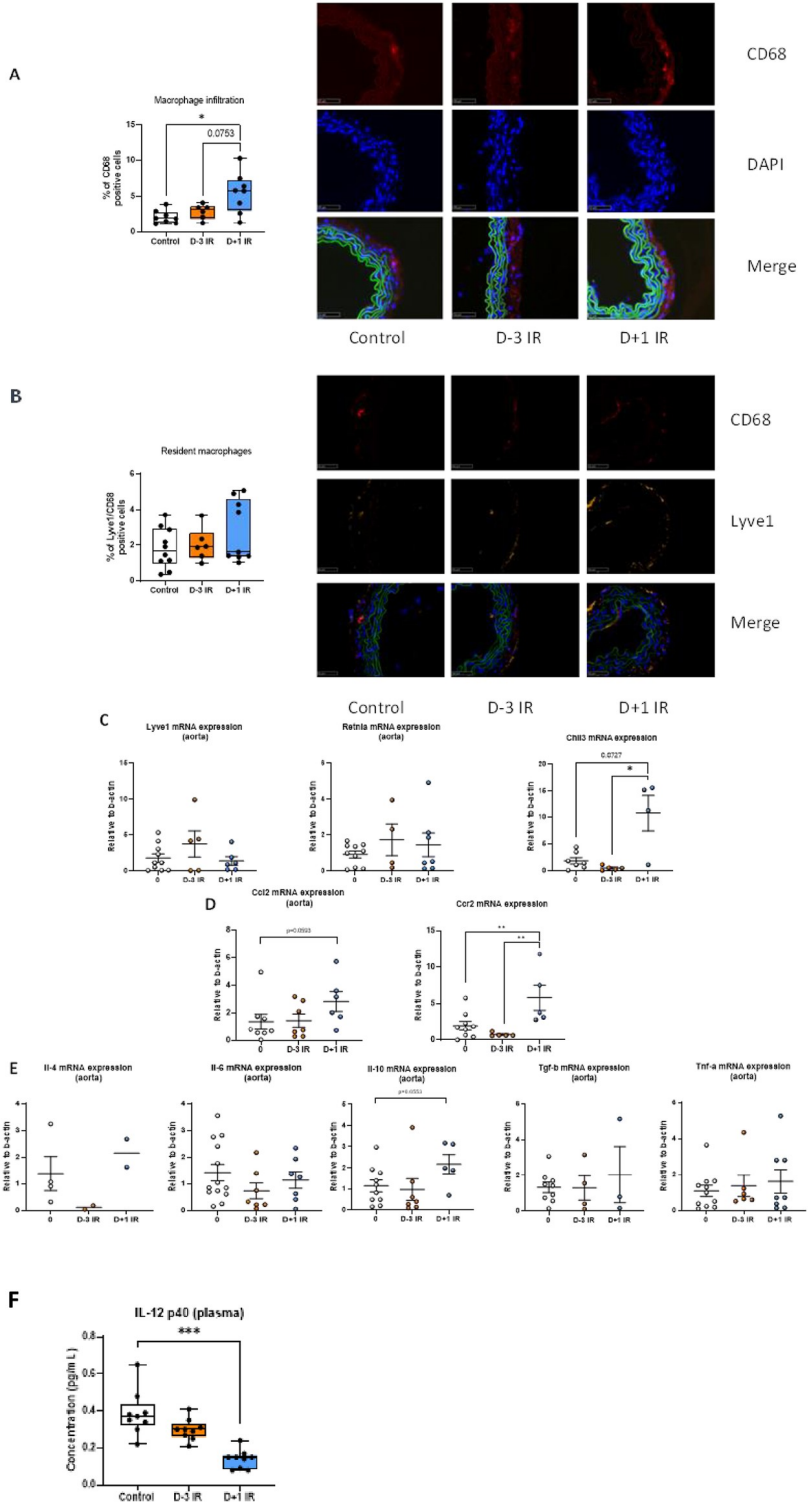
**A**, quantification of macrophage infiltration as a percentage of CD68-positive cells in the aorta at D7 (N = 6 to 8 mice per group). **B**, quantification of macrophage resident as a percentage of double-positive Lyve 1/CD68 cells in the aorta at D7 (N = 6 to 8 mice per group). **C**, quantification of *Ccl2*, and *Ccr2* mRNA expression in the aorta at day 7 by RT-qPCR (N = 5 to 8 mice per group). **D**, quantification of *Lyve1*, *Chi3l3*, and *Retnla* mRNA expression in the aorta at day 7 by RT-qPCR (N = 4 to 8 mice per group). **E**, quantification of *Il*-*6*, *Il-10*, *Tgf-β* and *Tnf-α* mRNA expression in the aorta at day 7 by RT-qPCR (N = 4 to 11 mice per group). **F**, quantification of cytokines *IL-12 p40* in plasma by Bio-Plex assay at D7 (N = 9 mice per group).

Given that immune cells participate in the pathophysiology of AAA development, we next investigated the impact of irradiation on local inflammation. First, we quantified the number of *CD68-positive* macrophages in aorta sections and evidenced a significant increase in the Day+1 group (Fig 5A) with no difference regarding Lyve1 expression, which is a marker of tissue resident macrophages. (Fig 5B). However, *Ccr2* and *Ccl2* mRNA levels were increased in the aorta of Day+1 group strongly suggesting that local macrophages were derived from infiltrating monocytes (Fig 5C). The increased number of macrophages associated with attenuation of AAA pathology induced by irradiation prompted us to investigate markers of macrophage profile and local inflammatory responses. We found an increase in the expression of *Chi3l3 gene* an M2-like marker in the Day+1 group, but no significant difference regarding *Lyve1* and *Retnla* mRNA levels (Fig 5D). Finally, increased levels of *Il-10* mRNA in the aorta of the Day+1 group strongly suggested an M2-like polarization of macrophage in this group (Fig 5E). The plasma cytokine profile was characterized by Bio-Plex. Unfortunately, almost all cytokines were undetectable (S3 Fig). We only found a significant reduction in Il-12 p40 levels in the Day+1 group compared to non-irradiated controls (Fig 5F).

### Irradiation induces ECM modifications within the vascular wall

**Fig 6:**
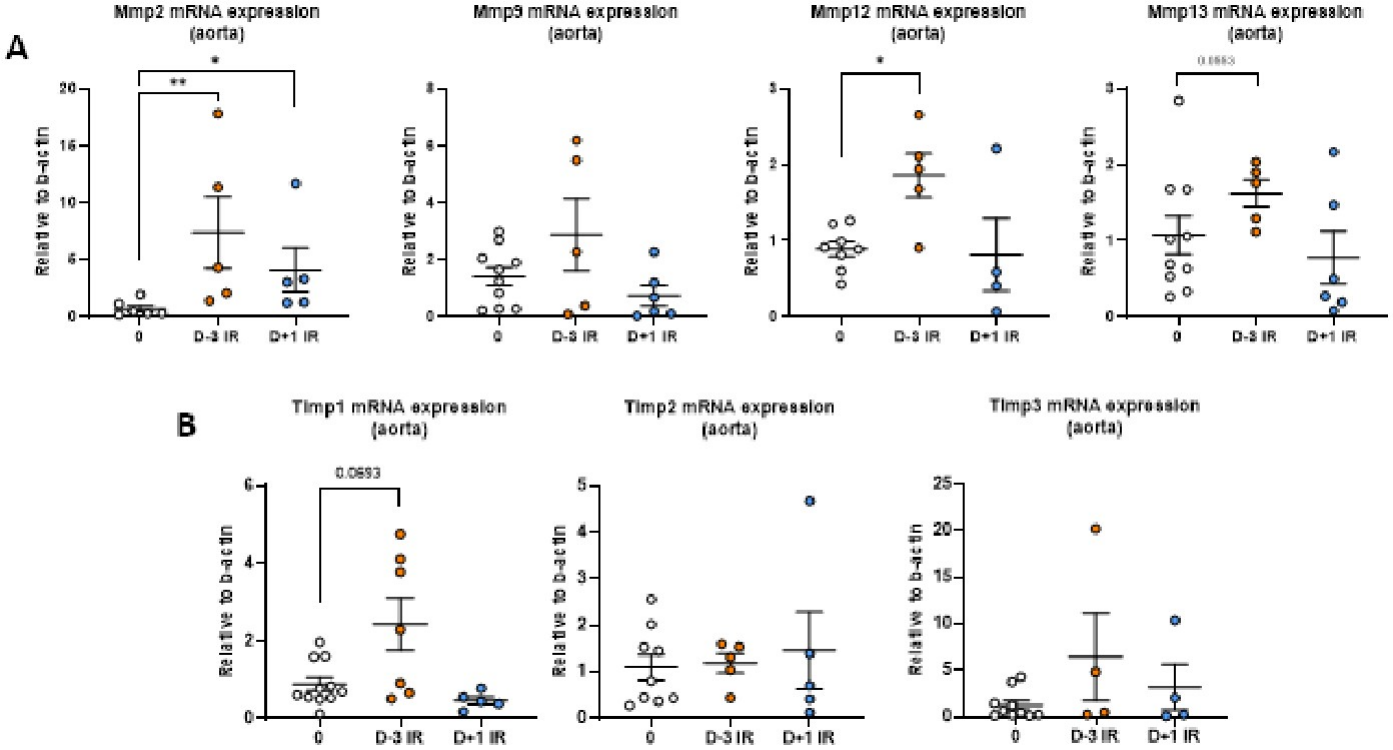
**A**, quantification of *Mmp2*, *Mmp9*, *Mmp12*, and *Mmp13* mRNA expression in the aorta at day 7 by RT-qPCR (Taqman) (N = 5 to 9 mice per group). **B**, quantification of *Timp1*, *Timp2*, and *Timp3* mRNA expression in the aorta at day 7 by RT-qPCR (Taqman) (N = 5 to 9 mice per group).

Next, we investigated protease gene expression in the aorta, proteases being key actors of remodeling and regulated by inflammation. Using qPCR, we found an increase in *Mmp-2*, *Mmp-12*, and *Mmp-13* mRNA levels in the aorta from the Day-3 irradiated group when compared to the non-irradiated group and an up-regulation of *Mmp-2* gene expression in the Day+1 group. (Fig 6A). At the same time, TIMP1, a powerful inhibitor of MMPs, was upregulated in the day+3 group compared to the non-irradiated controls. (Fig 6B).

### Global transcriptomic analysis

**Fig 7:**
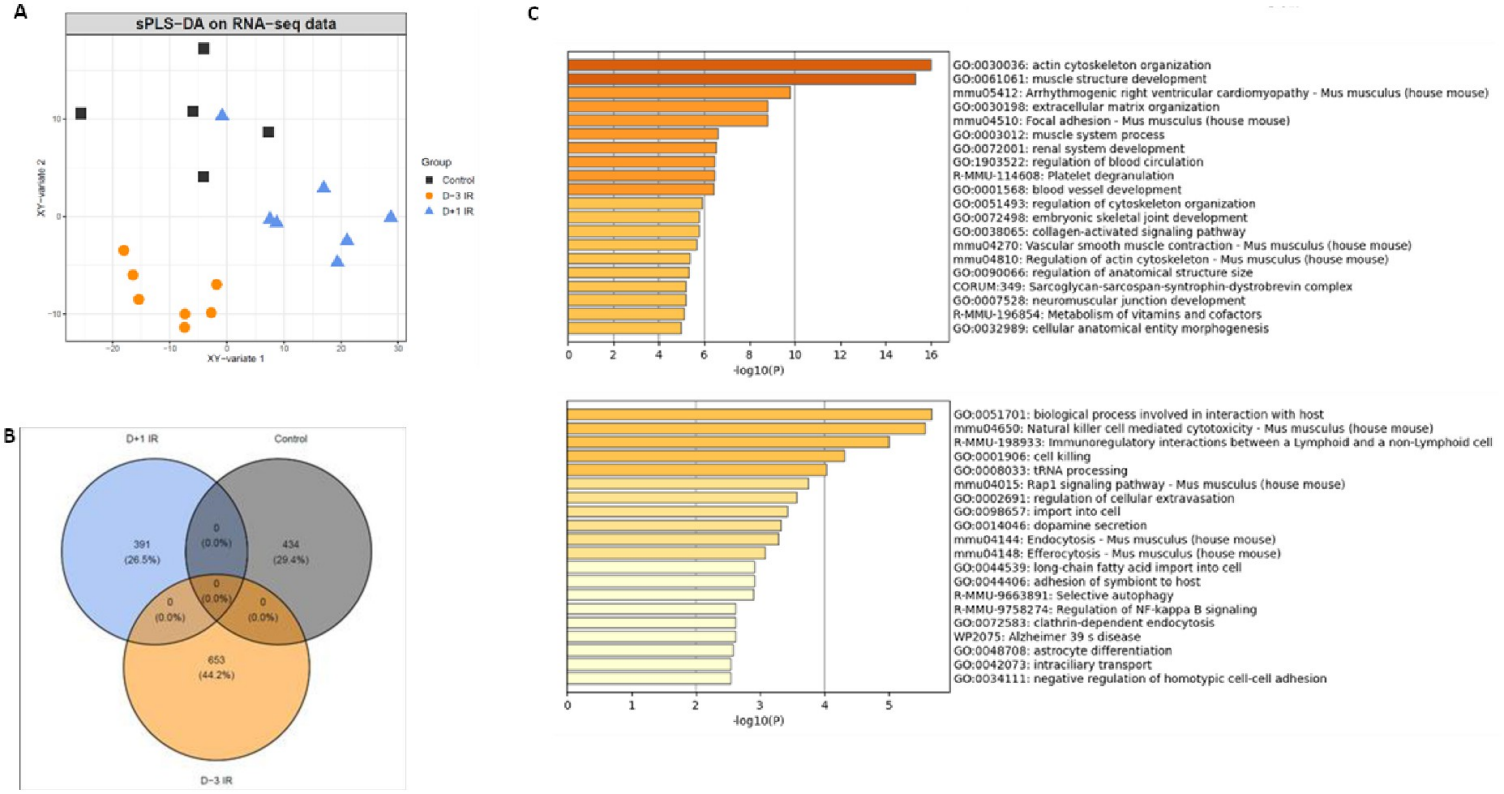
**A**, sPLS-DA on RNA-seq data (N = 5 to 7 mice per group). **B**, Venn diagram of genes explaining the differences between groups. **C**, Enrichment of genes explaining the first component (**top**) and the second component (**bottom**) (stability>70%, 1000 iterations).

Bulk RNA-seq sequencing was performed on the aorta from the three groups. Supervised sPLS-DA analysis discriminated each group based on two principal components (Fig 7A). A Venn diagram was constructed to identify the genes that best explained the clustering of each group within the sPLS-DA, selecting the top-ranking stable variables based to 2000 variable into selection analysis. 434 genes distinguished the non-irradiated control group, 391 genes distinguished the Day+1 IR irradiated group, and 653 genes distinguished the Day-3 IR irradiated group. No gene was shared among these three groups, suggesting different underlying pathways (Fig 7B). Enrichment analysis was performed on the two principal components. Only genes with a stability exceeding 70% were utilized for the enrichment process (S4 Fig). The first component consisted of genes primarily involved in the organization of actin cytoskeleton, ECM organization, SMC contraction, or cell adhesion. The second component was primarily composed of genes related to immunity. As shown in Fig 7C, we found that irradiation affected pathways related to the extracellular matrix, such as “actin cytoskeleton organization”, “extracellular matrix organization”, “collagen-activated signaling pathway”, “regulation of actin cytoskeleton” and also inflammatory pathways such as “Natural killer mediated cytotoxicity”, “immunoregulatory interactions between a Lymphoid and a non-Lymphoid cell“, “cell killing” or “regulation of NF-kappa B signaling”.

## Discussion

In this study, we showed that a single moderate dose of X-ray irradiation protects against experimental AAA formation. Irradiation was protective but the underlying molecular and cellular mechanisms differed with the time of irradiation. Irradiation before AngII infusion was associated with a phenotypic switch of SMCs while irradiation after AngII infusion was mainly associated with a modulation of inflammatory responses.

Here, we used a murine model for AAA with AngII infusion in hypercholesterolemic Apoe^-/-^ mice during 7 and 28 days. This widely recognized and documented model [22] was chosen for its ability to yield a robust phenotype, closely resembling human AAA development and providing valuable insights into its pathophysiological mechanisms. Indeed, AngII pathways were linked to human AAA pathophysiology and hypercholesterolemia recapitulated frequent co-morbidities and cardiovascular risks observed in AAA patients. We found a significant AAA reduction in irradiated animals but no difference in mortality. Such a discrepancy may be due, at least in part, to the cause of sudden death in AngII infused mice which is due to aneurysm rupture or mechanical dissection. Unfortunately, it was very difficult to differentiate these mechanisms during necropsy. Overall, our results are promising because we found that irradiation significantly reduced aorta dilation and it is well known that aorta diameter is strongly correlated with aorta wall tension and risk of rupture [23].

The common vasculo-protective mechanism we identified in the Day-3 and Day+1 groups was ECM remodeling and fibrosis with a significant increase in collagen1 content in the adventitia [24]. The cell source of collagen needs to be clarified and could be VSMC, fibroblasts or myofibroblasts. Unfortunately, distinction between these subsets of vascular cells remains challenging, with no fully specific markers [25]. Radio-induced fibrosis is a well-documented complication following radiotherapy in healthy tissue [26] but in our model radio-induced fibrosis was observed at a shorter time and a lower dose than previously described in the literature. The pathological context of the animal model used, coupled with the systemic inflammation induced by AngII, may support these findings.

### Irradiation before AngII infusion

When irradiation was done before AngII infusion, we mainly observed changes in VSMC phenotype but no clear inflammatory signature. This may be due to the fact that irradiation was done before AngII infusion, in a non-pathological context, in the absence of systemic inflammation. In this group, the reduction in alpha-SMA content in the aortic wall without any difference in cell number highly suggests changes in the VSMC phenotype and a loss of contractile phenotype. Increased *Lama5* and *Mmp2* mRNA levels supported this hypothesis. Lama5 is more associated with the epithelial and fibroblast signature [27, 28]. A dedifferentiation of VSMCs toward a myofibroblast-like secretory phenotype could explain the increased Collagen I content found in the aortic tissue [29]. In addition, we found a non-significant increase in *Tgf-β R1* gene expression which is also involved in the control of VSMC phenotype [30].

*Ex vivo* experiments also suggested that irradiation (1 Gy) impacted on the VSMC phenotype with increased proliferation as well as increased migration. The consequences of irradiation on VSMC functions may vary according to the dose and the experimental protocol. Previous works reported that gamma rays at doses of 10 Gy and 20 Gy on VSMCs reduced both proliferation and migration and promoted a phenotypic shift from a secretory to a contractile phenotype [31]. Additional analyses would be necessary to confirm the phenotypic switch of VSCM with analysis of pathways involved in VSMC plasticity such as myocardin and KLF4 [32].

In the Day-3 group, we observed an increase in collagen content in the aorta but also an increase in gene expression of several MMPs such as *Mmp-2*, *Mmp-12*, and *Mmp-13*. Several experimental works have identified their pathogenic role in the pathophysiology of AAA [33–35]. The role of *Mmp-12* is still currently controversial, with a recent study reporting vascular protection in *Mmp12* deficient mice [36]. Of note, gene expression does not systematically reflect protein levels and/or protease activity [37]. Additional experimentation should be done to directly quantify MMP activity with zymography. Finally, we also found an increase in *Timp-1* gene expression in the Day-3 group. TIMP1 is a powerful inhibitor of MMP activity [38], underlining that the impact of irradiation on MMP activity must be evaluated at the enzymatic level before conclusion.

### Irradiation after AngII infusion

In the group of mice irradiated 1 day after AngII infusion, we mainly identified inflammatory changes. At this timepoint, previous groups have shown that AngII quickly induced systemic and vascular inflammatory responses with acute activation and mobilization of monocytes into the blood stream followed by their recruitment into the aortic wall [39]. Interestingly, we found more macrophages in the aortic wall of Day+1 mice. Higher *Ccr2* and *Ccl2* mRNA levels with no difference in Lyve1 strongly suggest that macrophages were derived from infiltrating monocytes [40]. A recent study investigating the impact of gamma irradiation on experimental atherosclerosis demonstrated consistent findings, with increased macrophage content in atherosclerotic plaques and elevated levels of CCL2, KC and MIP-2 [41]. We did not analyze circulating monocytes in our work but in a previous work we have shown that high dose irradiation (1000 mGy) induced a reduction of blood Lyc6high monocytes but had no impact at lower doses [12]. Transcriptomic analysis suggested that macrophages had an anti-inflammatory profile as a gene expression of *Chi3l3*, an M2-like marker, and *IL-10* mRNA levels were increased [42]. Additional analyses would be required to confirm this phenotype. In the same way, we found a reduction in the plasma levels of IL-12 p40, a cytokine whose levels negatively correlate with IL-10 levels [43, 44]. IL-10 has been identified as a protective cytokine in the pathophysiology of AAA. IL-10-knockout mice displayed increased susceptibility to AngII-induced AAA formation and rupture [45]. Conversely, overexpression of IL-10 mitigates AAA [46]. The vasculoprotective mechanism of IL-10 is not completely understood but involved T cell modulation and collagen production [47].

## Conclusion

Acute irradiation with moderate doses of X-rays protects against experimental AAA formation. Irradiation was protective but the underlying molecular and cellular mechanisms were differed with the time of irradiation. Irradiation before Ang II infusion was associated with a phenotypic switch of VSMC while irradiation after Ang II infusion was associated with an anti-inflammatory signature. Our results showed that for the same pathology, exposure to ionizing radiation can lead to different mechanisms depending on the pathological context at the time of irradiation. Our results need confirmation but could be interest from on a therapeutic perspective if vascular protection is confirmed at lower doses or after local vascular exposure at moderate dose.

## Supporting information

S1 Fig Principal component analysis (PCA) on RNA-seq data. (PPTX)

S2 Fig Least-squares discriminant analysis (PLS-DA) on RNA-seq data. (PPTX)

S3 Fig Quantification of out-of-range cytokines in plasma using the Bio-Plex assay at Day 7. (PPTX)

S4 Fig Gene stability in the two components of sPLS-DA analysis. Only genes above the 70% threshold were used for enrichment.
(PPTX)

S1 Dataset Detailed individual data for each figure. (XLSX)

## Abbreviations

AAA: Abdominal Aortic Aneurysm
AngII: Angiotensin
CVD: Cardiovascular Diseases
ECM: Extracellular Matrix
PCA: Principal Component Analysis
PEA: Pathway Enrichment Analysis
PLS-DA: Partial Least-Squares Discriminant Analysis
SARRP: Small Animal Radiation Research Platform
VSMC: Vascular Smooth Muscle Cells
