# Supplementary figures and images for: Effects of moderate doses of ionizing radiation on experimental abdominal aortic aneurysm
Source: PLoS One. 2024 Aug 1;19(8):e0308273. doi: 10.1371/journal.pone.0308273 (PMC11293671; doi:10.1371/journal.pone.0308273)

## Slide 1
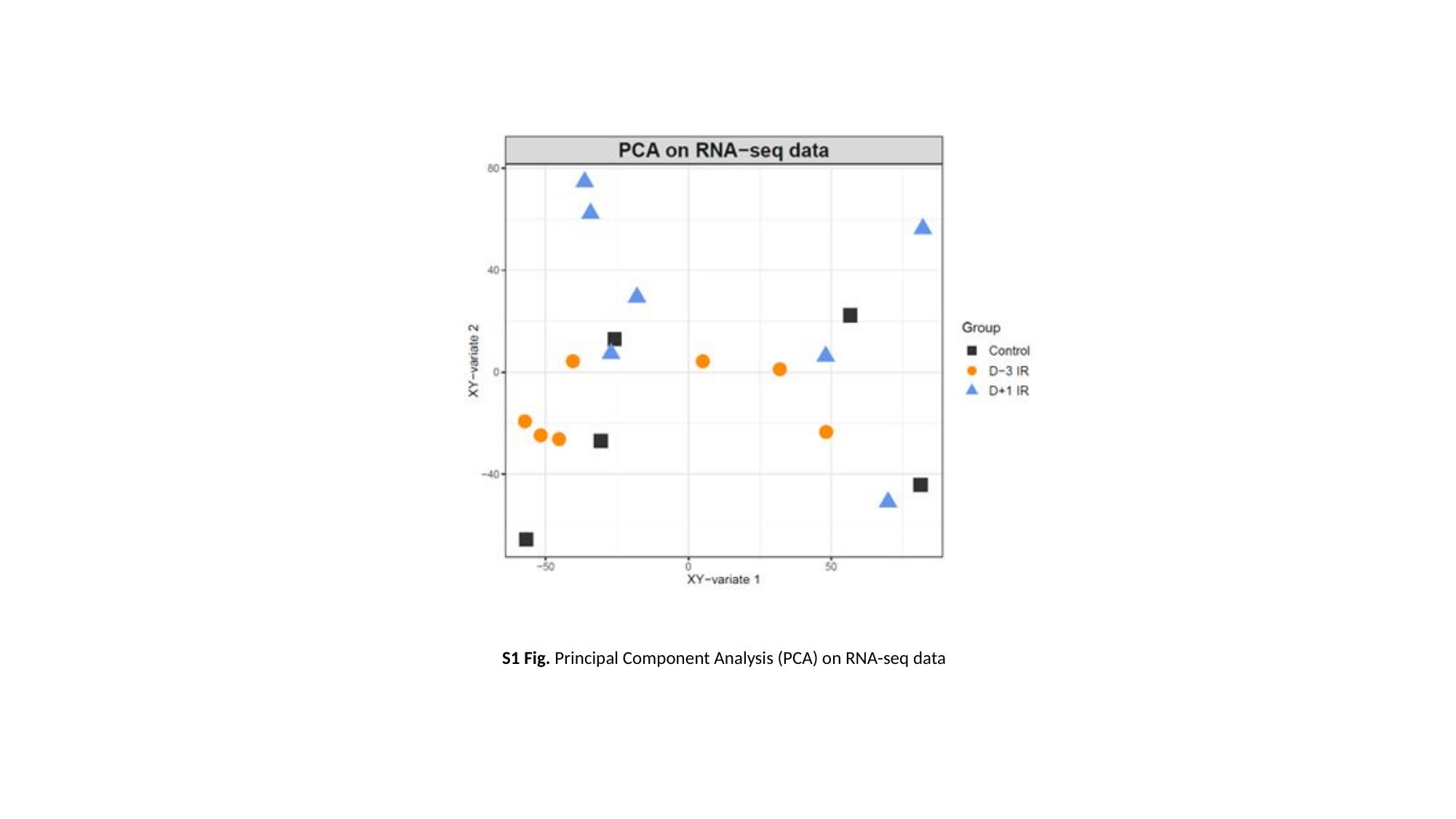

S1 Fig. Principal Component Analysis (PCA) on RNA-seq data

Supplement: S1 Fig — (PPTX) [file pone.0308273.s001.pptx]

## Slide 1
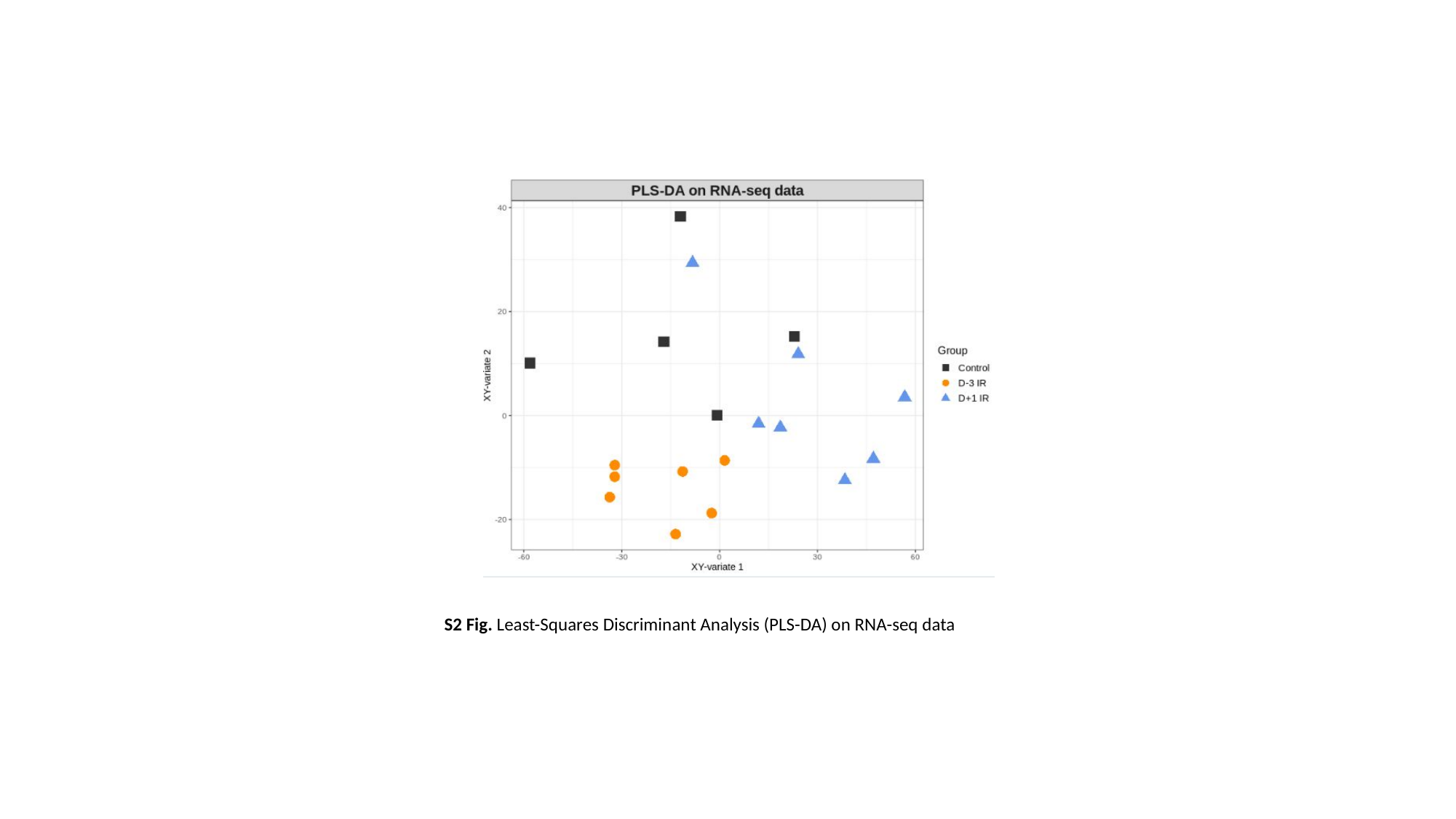

S2 Fig. Least-Squares Discriminant Analysis (PLS-DA) on RNA-seq data

Supplement: S2 Fig — (PPTX) [file pone.0308273.s002.pptx]

## Slide 1
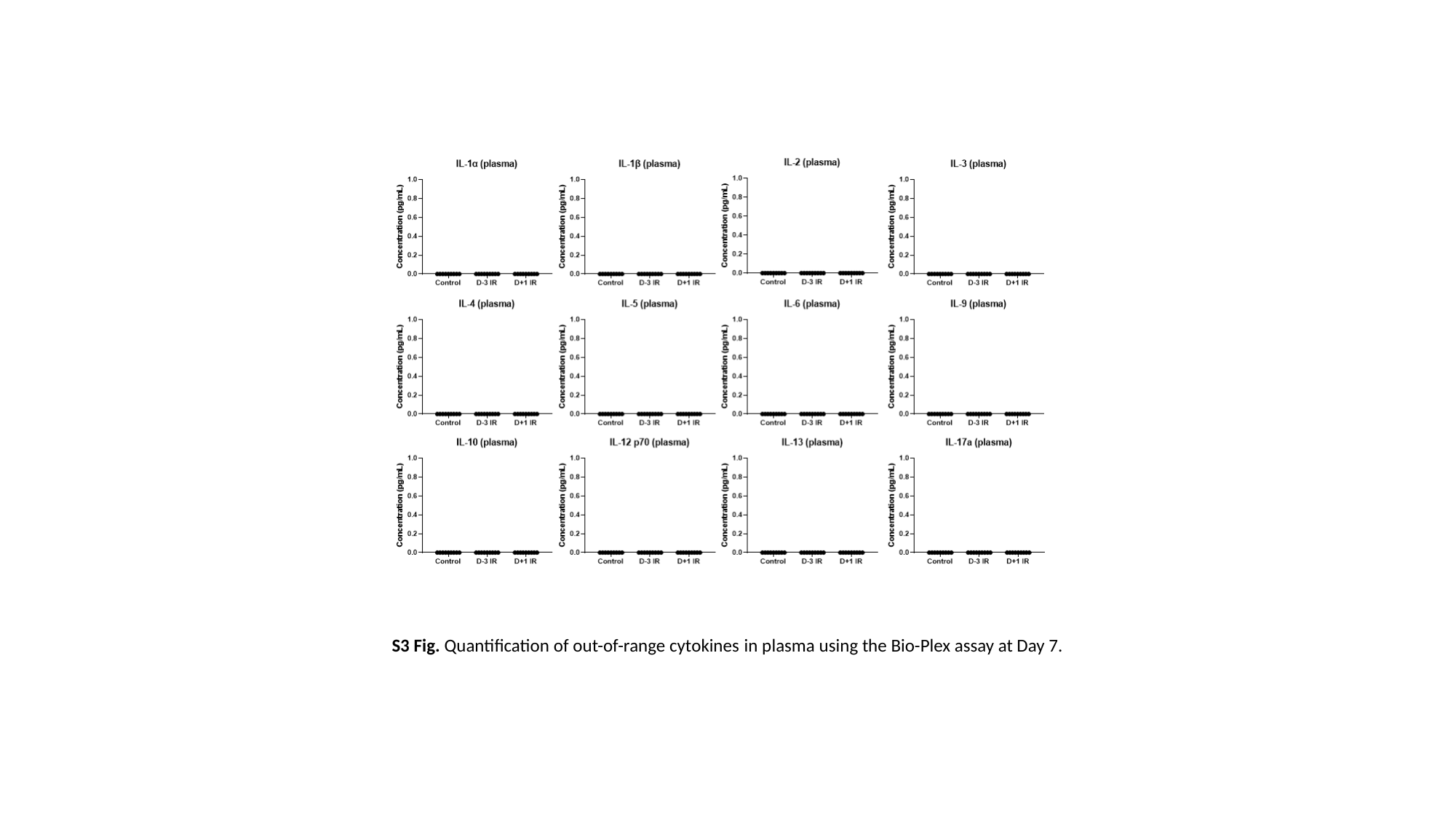

S3 Fig. Quantification of out-of-range cytokines in plasma using the Bio-Plex assay at Day 7.

Supplement: S3 Fig — (PPTX) [file pone.0308273.s003.pptx]
