# Supplementary material for: Effects of moderate doses of ionizing radiation on experimental abdominal aortic aneurysm
Source: PLoS One. 2024 Aug 1;19(8):e0308273. doi: 10.1371/journal.pone.0308273 (PMC11293671; doi:10.1371/journal.pone.0308273)
Supplement: S4 Fig — Only genes above the 70% threshold were used for enrichment. (PPTX) [file pone.0308273.s004.pptx]

## Slide 1
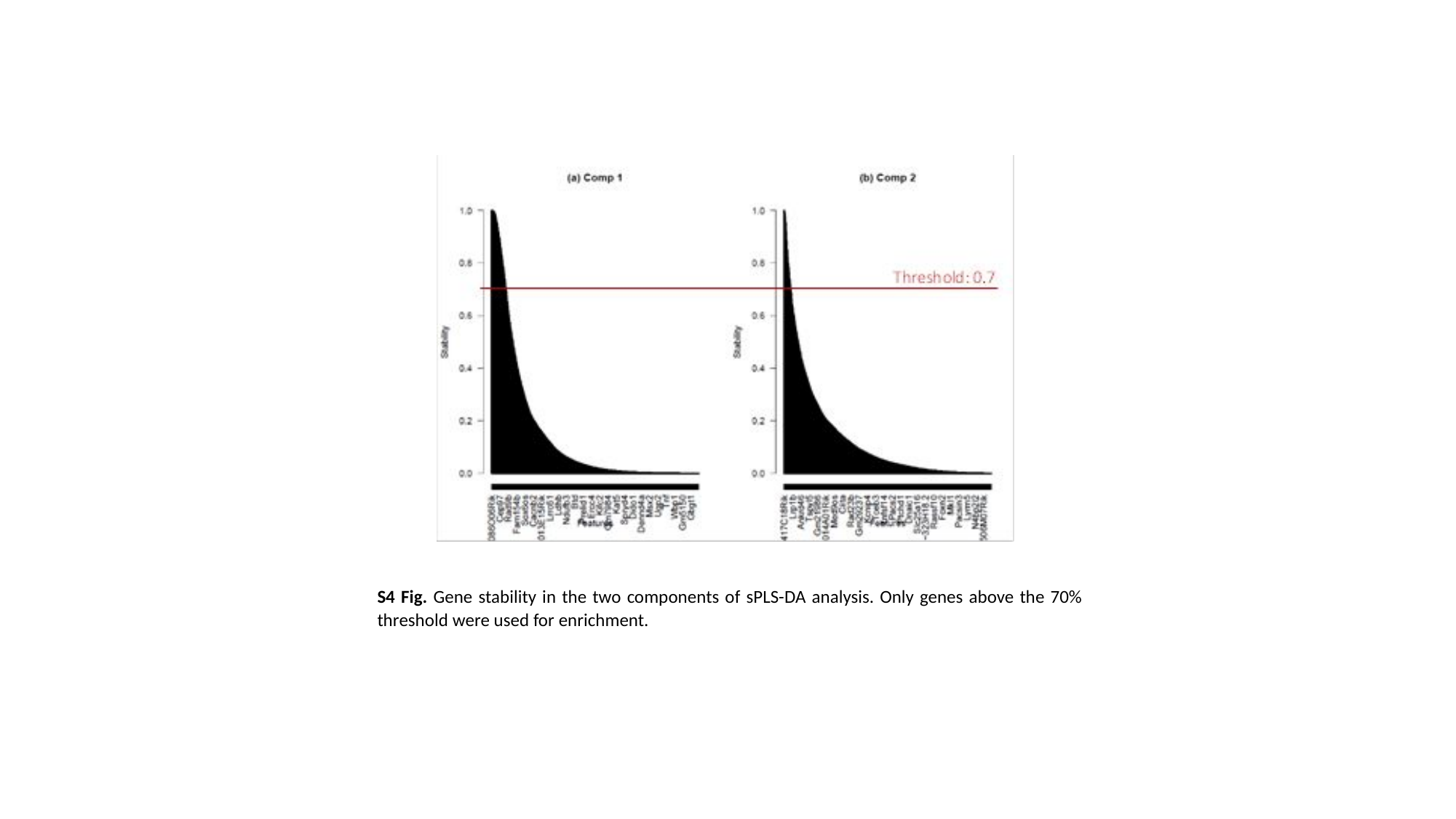

S4 Fig. Gene stability in the two components of sPLS-DA analysis. Only genes above the 70% threshold were used for enrichment.
