# Supplementary material for: Effects of moderate doses of ionizing radiation on experimental abdominal aortic aneurysm
Source: PLoS One. 2024 Aug 1;19(8):e0308273. doi: 10.1371/journal.pone.0308273 (PMC11293671; doi:10.1371/journal.pone.0308273)
Supplement: Attachment — Submitted filename: Response to reviewers.docx [file pone.0308273.s006.docx]

**Responses to comments**

**Editor Comments:**
In the protocol for vascular smooth muscle cells, it is unclear if the endothelium was removed. If not, there may be potential confounding effects of ECs in culture.

*Response: In our experimental protocol, we did not remove the endothelium, however the culture medium (DMEM F12 (D0697, Sigma-Aldrich) + 20% fetal bovine serum (FBS) (A5256701, Gibco)) promotes survival and proliferation of VSMCs but not endothelial cells. In addition, after several cell’s subculturing (between 3 or 5), αSMA immunostaining confirmed homogeneous VSMC population. This point has been clarified in the methods section.*

Total body irradiation protocol could be improved by demonstrating its effects on baseline macrophage/monocyte activity. This is because irradiation has impact on depletion of monocytes/macrophages in mice.

*Response: We would like to thank the editor for this valid comment. In a previous study, we have shown that high dose irradiation (1000 mGy) induced a reduction of blood Lyc6high monocytes (Rey et al, 2021) but had no impact on monocytes at lower doses. This point has been clarified in the discussion section.*

Figures were mislabeled (eg. In the results,… “Ang II infusion increased systolic blood pressure (figure 1A)”, however, figure 1A does not indicate this).

*Responses: The modifications have been done accordingly*

Many figures were very blurry and of low resolution. Finer details in the confocal images, including scale bars, are not visible. Also, some of the graphs (e.g., Figs 4E and 7A) have very tiny text on the x and y axes. This text should be made much bigger, so it is easily readable.

*Response: The modifications have been done accordingly*

Sample sizes are not described in the paper. Although the replicates are shown in the graphs, which conveys transparency of data, the N number seems to change between adjacent experiments (e.g. Figure 4B seems to have random numbers of samples for each marker they looked at.). The color of the graphs is also inconsistent. Making graphs of the same type with the same groups with the same colour helps for continuity from experiment to experiment while also being more visually appealing.

*Response: Sample sizes and replicates are now shown in the graphs. We used a color code: model validation in black and white. For the subsequent protocol on D-3, it's in orange, and for the protocol on D+1, it's in blue. Most of the graphs are in box plots, except for the PCRs.*

Reviewer #2: An aortic aneurysm (AAA) is a balloon-like bulge in the aorta which is life threatening cardiovascular disease. However, the treatment for AAA is not fully developed. Riazi et al., examined the protective role of irradiation on AAA development. Authors found that irradiation decreased AAA formation by modulating VSMC phenotype and inflammation. This is an important study, but the reviewer has the following comments.

What is the possible mechanism of irradiation-mediated changes VSMC phenotype changes or collagen content? Please include in the discussion section.

*Response: We would like to thank the reviewer for this comment. Potential mechanisms for irradiation-mediated changes in VSMC phenotype have been discussed in the discussion section (Pages 23 and 24).*

In figure 1B, Authors should provide the absolute value of systolic blood pressure (expressed as mm/Hg).

*Response: the modifications have been done accordingly*

Maximal abdominal aortic diameter shown in Fig 1D. Please provide the representative whole aorta picture from PBS and Ang II infused mice which showed abdominal aortic dilation.

*Response: As suggested representative pictures of aortas from PBS or angioII treated mice have been added in figure 1.*

In fig 3, authors showed Ang II induced vascular remodeling at Day 7. Since the vascular remodeling and dilation happened at a later stage. What is the rationale to use Day 7 sample for vascular remodeling in Fig 3.

*Response : Our strategy was to investigate matrix remodeling before aneurysm formation and complications. At later stages, aneurysm severity impacts a lot on matrix remodeling and represents a confounder. In other words, the more severe aneurysms are characterized by higher remodeling. That’s the reason why in all our studies (ex: Giraud et al. Cardiovasc Res 2015; Vandestienne et al. JCI 2021), we standardized the analysis of extracellular matrix at day 3 or 7.*

In fig 3A, authors showed Ang II-infused mice (as control) and irradiation. Ang II infused mice should disintegrate the elastin layer and leads to balloon-like structure. But the representative picture showed intact elastin as well as no dilation after Ang II infusion. Please explain the inconsistency with fig 2. Authors should include control (PBS) group.

*Response: As suggested we have inserted more representative pictures.*

What is the rationale to use control VSMC in Fig 4C-F. How does this data correlate with AAA?
*Response: The objective of these ex vivo tests was to describe the impact of irradiation on VSMCs phenotype. These experiments have been done because we observed decrease a-SMA content in the aortic media of irradiated mice suggesting a modification of cell profile. Analysis of proliferation and/or migration is helpful to evaluate VSMC phenotypic switch after irradiation.*

Plasma levels of cytokines are not detectable in Supplemental fig 3. Ang II infused mice or AAA mice should increase cytokines level in plasma. Why were authors not able to detect?

*Responses : Cytokines were quantified using the Bioplex method at day 7. The absence of detection could be due to the method of quantification (which potentially lacks) sensitivity or could be explained by the timing. Indeed, AngII infusion induces acute systemic responses within the first 3 days but after this early response, despite chronic infusion systemic inflammatory response decreases. In our hands, we have previously observed that monocyte count in the blood increased at day 3 after angioII and next returned to baseline. However, inflammatory responses in the aortic wall remains active during longer period.*
