# Supplementary material for: Effects of moderate doses of ionizing radiation on experimental abdominal aortic aneurysm
Source: PLoS One. 2024 Aug 1;19(8):e0308273. doi: 10.1371/journal.pone.0308273 (PMC11293671; doi:10.1371/journal.pone.0308273)
Supplement: Peer review history

# Peer review: Decision Letter 0

**DOI:** 10.1371/journal.pone.0308273.r001

**Authors:** Venkateswaran Subramanian

2 Apr 2024

PONE-D-24-09315Effects of moderate doses of ionizing radiation on experimental abdominal aortic aneurysmPLOS ONE

Dear Dr. Ait-Oufella,

Thank you for submitting your manuscript to PLOS ONE. After careful consideration, we feel that it has merit but does not fully meet PLOS ONE’s publication criteria as it currently stands. Therefore, we invite you to submit a revised version of the manuscript that addresses the points raised during the review process.

Please submit your revised manuscript by May 17 2024 11:59PM. If you will need more time than this to complete your revisions, please reply to this message or contact the journal office at plosone@plos.org. When you're ready to submit your revision, log on to https://www.editorialmanager.com/pone/ and select the 'Submissions Needing Revision' folder to locate your manuscript file.

Please include the following items when submitting your revised manuscript:

- A rebuttal letter that responds to each point raised by the academic editor and reviewer(s). You should upload this letter as a separate file labeled 'Response to Reviewers'.
- A marked-up copy of your manuscript that highlights changes made to the original version. You should upload this as a separate file labeled 'Revised Manuscript with Track Changes'.
- An unmarked version of your revised paper without tracked changes. You should upload this as a separate file labeled 'Manuscript'.

If you would like to make changes to your financial disclosure, please include your updated statement in your cover letter. Guidelines for resubmitting your figure files are available below the reviewer comments at the end of this letter.

If applicable, we recommend that you deposit your laboratory protocols in protocols.io to enhance the reproducibility of your results. Protocols.io assigns your protocol its own identifier (DOI) so that it can be cited independently in the future. For instructions see: https://journals.plos.org/plosone/s/submission-guidelines#loc-laboratory-protocols. Additionally, PLOS ONE offers an option for publishing peer-reviewed Lab Protocol articles, which describe protocols hosted on protocols.io. Read more information on sharing protocols at https://plos.org/protocols?utm_medium=editorial-email&utm_source=authorletters&utm_campaign=protocols.

We look forward to receiving your revised manuscript.

Kind regards,

Venkateswaran Subramanian, Ph.D

Academic Editor

PLOS ONE

Journal Requirements:

When submitting your revision, we need you to address these additional requirements.

1. Please ensure that your manuscript meets PLOS ONE's style requirements, including those for file naming. The PLOS ONE style templates can be found at 

https://journals.plos.org/plosone/s/file?id=wjVg/PLOSOne_formatting_sample_main_body.pdf and 

https://journals.plos.org/plosone/s/file?id=ba62/PLOSOne_formatting_sample_title_authors_affiliations.pdf

2. To comply with PLOS ONE submissions requirements, in your Methods section, please provide additional information regarding the experiments involving animals and ensure you have included details on methods of sacrifice, and efforts to alleviate suffering.

3. We note that the grant information you provided in the ‘Funding Information’ and ‘Financial Disclosure’ sections do not match. 

When you resubmit, please ensure that you provide the correct grant numbers for the awards you received for your study in the ‘Funding Information’ section.

4. Thank you for stating the following financial disclosure: "Electricité de France"

Please state what role the funders took in the study.  If the funders had no role, please state: "The funders had no role in study design, data collection and analysis, decision to publish, or preparation of the manuscript."

If this statement is not correct you must amend it as needed. 

Please include this amended Role of Funder statement in your cover letter; we will change the online submission form on your behalf.

5. Thank you for stating the following in your Competing Interests section: "NO authors have competing interests"

Please complete your Competing Interests on the online submission form to state any Competing Interests. If you have no competing interests, please state "The authors have declared that no competing interests exist.", as detailed online in our guide for authors at http://journals.plos.org/plosone/s/submit-now 

This information should be included in your cover letter; we will change the online submission form on your behalf.

6. Please include captions for your Supporting Information files at the end of your manuscript, and update any in-text citations to match accordingly. Please see our Supporting Information guidelines for more information: http://journals.plos.org/plosone/s/supporting-information. 

**Additional Editor Comments:**

In addition to the reviewer’s comments,

- Your manuscript requires thorough editing of the English language and style to ensure it is grammatically correct and error-free.  
- The resolution of figures and images needs to be improved.
- Errors in figure labeling should be fixed

[Note: HTML markup is below. Please do not edit.]

Reviewers' comments:

Reviewer's Responses to Questions

**Comments to the Author**

1. Is the manuscript technically sound, and do the data support the conclusions?

The manuscript must describe a technically sound piece of scientific research with data that supports the conclusions. Experiments must have been conducted rigorously, with appropriate controls, replication, and sample sizes. The conclusions must be drawn appropriately based on the data presented. 

Reviewer #1: Yes

Reviewer #2: No

**********

2. Has the statistical analysis been performed appropriately and rigorously? 

Reviewer #1: Yes

Reviewer #2: I Don't Know

**********

3. Have the authors made all data underlying the findings in their manuscript fully available?

The PLOS Data policy requires authors to make all data underlying the findings described in their manuscript fully available without restriction, with rare exception (please refer to the Data Availability Statement in the manuscript PDF file). The data should be provided as part of the manuscript or its supporting information, or deposited to a public repository. For example, in addition to summary statistics, the data points behind means, medians and variance measures should be available. If there are restrictions on publicly sharing data—e.g. participant privacy or use of data from a third party—those must be specified.

Reviewer #1: Yes

Reviewer #2: Yes

**********

4. Is the manuscript presented in an intelligible fashion and written in standard English?

PLOS ONE does not copyedit accepted manuscripts, so the language in submitted articles must be clear, correct, and unambiguous. Any typographical or grammatical errors should be corrected at revision, so please note any specific errors here.

Reviewer #1: No

Reviewer #2: No

**********

5. Review Comments to the Author

Please use the space provided to explain your answers to the questions above. You may also include additional comments for the author, including concerns about dual publication, research ethics, or publication ethics. (Please upload your review as an attachment if it exceeds 20,000 characters)

Reviewer #1: In the manuscript titled “Effects of moderate doses of ionizing radiation on experimental abdominal aortic aneurysm,” Riazi et al. attempted to explore the effects of moderate doses of ionizing radiation on abdominal aortic aneurysms. Ang II-infused ApoE-/- mice were irradiated either 3 days prior or 1 day following micro-osmotic pump surgery, which disrupted normal disease progression in both cases. Irradiation 3 days prior to Ang II infusion was associated with a phenotypic switch of vSMCs, and irradiation 1 day after Ang II infusion was associated with anti-inflammatory effects. Overall, this paper provides intriguing data but lacks mechanistic work to corroborate findings.

Concerns:

The document's content is understandable and the authors' message is clear, but the grammar needs significant improvement. It appears that the document may have been translated from French since some of the sentences have unusual word choices or singular/plural errors. Therefore, the document requires thorough editing to ensure that it is grammatically correct and free of errors.

In the protocol for vascular smooth muscle cells, it is unclear if the endothelium was removed. If not, there may be potential confounding effects of ECs in culture.

Total body irradiation protocol could be improved by demonstrating its effects on baseline macrophage/monocyte activity. This is because irradiation has impact on depletion of monocytes/macrophages in mice.

Figures were mislabeled (eg. In the results,… “Ang II infusion increased systolic blood pressure (figure 1A)”, however, figure 1A does not indicate this).

Many figures were very blurry and of low resolution. Finer details in the confocal images, including scale bars, are not visible. Also, some of the graphs (e.g., Figs 4E and 7A) have very tiny text on the x and y axes. This text should be made much bigger, so it is easily readable.

Sample sizes are not described in the paper. Although the replicates are shown in the graphs, which conveys transparency of data, the N number seems to change between adjacent experiments (e.g. Figure 4B seems to have random numbers of samples for each marker they looked at.). The color of the graphs is also inconsistent. Making graphs of the same type with the same groups with the same colour helps for continuity from experiment to experiment while also being more visually appealing.

Reviewer #2: An aortic aneurysm (AAA) is a balloon-like bulge in the aorta which is life threatening cardiovascular disease. However, the treatment for AAA is not fully developed. Riazi et al., examined the protective role of irradiation on AAA development. Authors found that irradiation decreased AAA formation by modulating VSMC phenotype and inflammation. This is an important study, but the reviewer has the following comments.

What is the possible mechanism of irradiation-mediated changes VSMC phenotype changes or collagen content? Please include in the discussion section.

In figure 1B, Authors should provide the absolute value of systolic blood pressure (expressed as mm/Hg). What is the meaning of normalized blood pressure? As same, what is meaning of heart weight/total weight (%) (Fig 1C). It may be ratio of heart weight/total weight.

Maximal abdominal aortic diameter shown in Fig 1D. Please provide the representative whole aorta picture from PBS and Ang II infused mice which showed abdominal aortic dilation.

In fig 3, authors showed Ang II induced vascular remodeling at Day 7. Since the vascular remodeling and dilation happened at a later stage. What is the rationale to use Day 7 sample for vascular remodeling in Fig 3.

In fig 3A, authors showed Ang II-infused mice (as control) and irradiation. Ang II infused mice should disintegrate the elastin layer and leads to balloon-like structure. But the representative picture showed intact elastin as well as no dilation after Ang II infusion. Please explain the inconsistency with fig 2. Authors should include control (PBS) group.

What is the rationale to use control VSMC in Fig 4C-F. How does this data correlate with AAA?

Plasma levels of cytokines are not detectable in Supplemental fig 3. Ang II infused mice or AAA mice should increase cytokines level in plasma. Why were authors not able to detect?

**********

6. PLOS authors have the option to publish the peer review history of their article (what does this mean?). If published, this will include your full peer review and any attached files.

If you choose “no”, your identity will remain anonymous but your review may still be made public.

**Do you want your identity to be public for this peer review?** For information about this choice, including consent withdrawal, please see our Privacy Policy.

Reviewer #1: No

Reviewer #2: No

**********

[NOTE: If reviewer comments were submitted as an attachment file, they will be attached to this email and accessible via the submission site. Please log into your account, locate the manuscript record, and check for the action link "View Attachments". If this link does not appear, there are no attachment files.]

While revising your submission, please upload your figure files to the Preflight Analysis and Conversion Engine (PACE) digital diagnostic tool, https://pacev2.apexcovantage.com/. PACE helps ensure that figures meet PLOS requirements. To use PACE, you must first register as a user. Registration is free. Then, login and navigate to the UPLOAD tab, where you will find detailed instructions on how to use the tool. If you encounter any issues or have any questions when using PACE, please email PLOS at figures@plos.org. Please note that Supporting Information files do not need this step.

# Author comment: Author response to Decision Letter 0

**DOI:** 10.1371/journal.pone.0308273.r002

7 Jun 2024

Responses to comments

Editor Comments:

In the protocol for vascular smooth muscle cells, it is unclear if the endothelium was removed. If not, there may be potential confounding effects of ECs in culture.

Response: In our experimental protocol, we did not remove the endothelium, however the culture medium (DMEM F12 (D0697, Sigma-Aldrich) + 20% fetal bovine serum (FBS) (A5256701, Gibco)) promotes survival and proliferation of VSMCs but not endothelial cells. In addition, after several cell’s subculturing (between 3 or 5), αSMA immunostaining confirmed homogeneous VSMC population. This point has been clarified in the methods section.

Total body irradiation protocol could be improved by demonstrating its effects on baseline macrophage/monocyte activity. This is because irradiation has impact on depletion of monocytes/macrophages in mice. 

Response: We would like to thank the editor for this valid comment. In a previous study, we have shown that high dose irradiation (1000 mGy) induced a reduction of blood Lyc6high monocytes (Rey et al, 2021) but had no impact on monocytes at lower doses. This point has been clarified in the discussion section. 

Figures were mislabeled (eg. In the results,… “Ang II infusion increased systolic blood pressure (figure 1A)”, however, figure 1A does not indicate this).

Responses: The modifications have been done accordingly

Many figures were very blurry and of low resolution. Finer details in the confocal images, including scale bars, are not visible. Also, some of the graphs (e.g., Figs 4E and 7A) have very tiny text on the x and y axes. This text should be made much bigger, so it is easily readable.

Response: The modifications have been done accordingly

Sample sizes are not described in the paper. Although the replicates are shown in the graphs, which conveys transparency of data, the N number seems to change between adjacent experiments (e.g. Figure 4B seems to have random numbers of samples for each marker they looked at.). The color of the graphs is also inconsistent. Making graphs of the same type with the same groups with the same colour helps for continuity from experiment to experiment while also being more visually appealing.

Response: Sample sizes and replicates are now shown in the graphs. We used a color code: model validation in black and white. For the subsequent protocol on D-3, it's in orange, and for the protocol on D+1, it's in blue. Most of the graphs are in box plots, except for the PCRs.

Reviewer #2: An aortic aneurysm (AAA) is a balloon-like bulge in the aorta which is life threatening cardiovascular disease. However, the treatment for AAA is not fully developed. Riazi et al., examined the protective role of irradiation on AAA development. Authors found that irradiation decreased AAA formation by modulating VSMC phenotype and inflammation. This is an important study, but the reviewer has the following comments.

What is the possible mechanism of irradiation-mediated changes VSMC phenotype changes or collagen content? Please include in the discussion section.

Response: We would like to thank the reviewer for this comment. Potential mechanisms for irradiation-mediated changes in VSMC phenotype have been discussed in the discussion section (Pages 23 and 24).

In figure 1B, Authors should provide the absolute value of systolic blood pressure (expressed as mm/Hg). 

Response: the modifications have been done accordingly

Maximal abdominal aortic diameter shown in Fig 1D. Please provide the representative whole aorta picture from PBS and Ang II infused mice which showed abdominal aortic dilation.

Response: As suggested representative pictures of aortas from PBS or angioII treated mice have been added in figure 1.

In fig 3, authors showed Ang II induced vascular remodeling at Day 7. Since the vascular remodeling and dilation happened at a later stage. What is the rationale to use Day 7 sample for vascular remodeling in Fig 3.

Response : Our strategy was to investigate matrix remodeling before aneurysm formation and complications. At later stages, aneurysm severity impacts a lot on matrix remodeling and represents a confounder. In other words, the more severe aneurysms are characterized by higher remodeling. That’s the reason why in all our studies (ex: Giraud et al. Cardiovasc Res 2015; Vandestienne et al. JCI 2021), we standardized the analysis of extracellular matrix at day 3 or 7.

In fig 3A, authors showed Ang II-infused mice (as control) and irradiation. Ang II infused mice should disintegrate the elastin layer and leads to balloon-like structure. But the representative picture showed intact elastin as well as no dilation after Ang II infusion. Please explain the inconsistency with fig 2. Authors should include control (PBS) group.

Response: As suggested we have inserted more representative pictures.

What is the rationale to use control VSMC in Fig 4C-F. How does this data correlate with AAA?

Response: The objective of these ex vivo tests was to describe the impact of irradiation on VSMCs phenotype. These experiments have been done because we observed decrease a-SMA content in the aortic media of irradiated mice suggesting a modification of cell profile. Analysis of proliferation and/or migration is helpful to evaluate VSMC phenotypic switch after irradiation.

Plasma levels of cytokines are not detectable in Supplemental fig 3. Ang II infused mice or AAA mice should increase cytokines level in plasma. Why were authors not able to detect?

Responses : Cytokines were quantified using the Bioplex method at day 7. The absence of detection could be due to the method of quantification (which potentially lacks) sensitivity or could be explained by the timing. Indeed, AngII infusion induces acute systemic responses within the first 3 days but after this early response, despite chronic infusion systemic inflammatory response decreases. In our hands, we have previously observed that monocyte count in the blood increased at day 3 after angioII and next returned to baseline. However, inflammatory responses in the aortic wall remains active during longer period.

Attachment Submitted filename: Response to reviewers.docx

# Peer review: Decision Letter 1

**DOI:** 10.1371/journal.pone.0308273.r003

**Authors:** Venkateswaran Subramanian

19 Jul 2024

Effects of moderate doses of ionizing radiation on experimental abdominal aortic aneurysm

PONE-D-24-09315R1

Dear Dr. Ait-Oufella,

We’re pleased to inform you that your manuscript has been judged scientifically suitable for publication and will be formally accepted for publication once it meets all outstanding technical requirements.

Within one week, you’ll receive an e-mail detailing the required amendments. When these have been addressed, you’ll receive a formal acceptance letter and your manuscript will be scheduled for publication.

An invoice will be generated when your article is formally accepted. Please note, if your institution has a publishing partnership with PLOS and your article meets the relevant criteria, all or part of your publication costs will be covered. Please make sure your user information is up-to-date by logging into Editorial Manager at Editorial Manager® and clicking the ‘Update My Information' link at the top of the page. If you have any questions relating to publication charges, please contact our Author Billing department directly at authorbilling@plos.org.

If your institution or institutions have a press office, please notify them about your upcoming paper to help maximize its impact. If they’ll be preparing press materials, please inform our press team as soon as possible -- no later than 48 hours after receiving the formal acceptance. Your manuscript will remain under strict press embargo until 2 pm Eastern Time on the date of publication. For more information, please contact onepress@plos.org.

Kind regards,

Venkateswaran Subramanian, Ph.D

Academic Editor

PLOS ONE

# Editor report: Acceptance letter

**DOI:** 10.1371/journal.pone.0308273.r004

**Authors:** Venkateswaran Subramanian

24 Jul 2024

PONE-D-24-09315R1 

PLOS ONE

Dear Dr. Ait-Oufella, 

I'm pleased to inform you that your manuscript has been deemed suitable for publication in PLOS ONE. Congratulations! Your manuscript is now being handed over to our production team.

At this stage, our production department will prepare your paper for publication. This includes ensuring the following:

* All references, tables, and figures are properly cited

* All relevant supporting information is included in the manuscript submission,

* There are no issues that prevent the paper from being properly typeset

If revisions are needed, the production department will contact you directly to resolve them. If no revisions are needed, you will receive an email when the publication date has been set. At this time, we do not offer pre-publication proofs to authors during production of the accepted work. Please keep in mind that we are working through a large volume of accepted articles, so please give us a few weeks to review your paper and let you know the next and final steps. 

Lastly, if your institution or institutions have a press office, please let them know about your upcoming paper now to help maximize its impact. If they'll be preparing press materials, please inform our press team within the next 48 hours. Your manuscript will remain under strict press embargo until 2 pm Eastern Time on the date of publication. For more information, please contact onepress@plos.org.

If we can help with anything else, please email us at customercare@plos.org.

Thank you for submitting your work to PLOS ONE and supporting open access. 

Kind regards, 

PLOS ONE Editorial Office Staff

on behalf of

Dr. Venkateswaran Subramanian 

Academic Editor

PLOS ONE
